# Cu-Substituted Sb_2_S_3_ Nanopowders—Structural, Microstructural and Optical Analyses

**DOI:** 10.3390/ijms27167406

**Published:** 2026-08-19

**Authors:** Nikola Ilić, Cristian Radu, Amelia Elena Bocirnea, Aurelian Catalin Galca, Ivana Validžić

**Affiliations:** 1 Department of Atomic Physics, Vinča Institute of Nuclear Sciences, National Institute of the Republic of Serbia, University of Belgrade, 11000 Belgrade, Serbia; 2 National Institute of Materials Physics, Atomistilor 405A, 077125 Magurele, Romania

**Keywords:** antimony sulfide, optical absorbers, CuSbS_2_, TEM, structural analysis, morphology

## Abstract

Cu(I)-doped Sb_2_S_3_ powders were synthesized using the hot-injection method, with the aim of reducing crystalline particle size and tailoring their morphology to make them more spherical and better suited for spray deposition as an absorber layer in photovoltaic devices. Structural and microstructural analyses confirmed the successful formation of elongated (rod-like) crystalline Sb_2_S_3_ particles. CuSbS_2_, itself a semiconducting material with a high light-absorption coefficient, formed in large amounts in Cu-doped systems, in the form of agglomerates of platelet-like particles. The third abundant phase was an amorphous phase present as spherical nanoparticles composed of uniformly distributed Sb, Cu and S. The presence of Cu decreased Sb_2_S_3_ particle size but did not significantly influence its morphology. Chlorides in the synthesis solution enabled faster crystallization than when acetates are the only present anionic species. When Cu, Sb and S sources were added during hot injection in stoichiometric amounts to form CuSbS_2_, this phase was not reached, and Cu_9_S_5_ and Cu_12_Sb_4_S_13_ were the dominant phases, indicating Cu-rich phases have a higher tendency to crystallize in specific conditions with an excess of antimony, and, potentially, sulfur is needed to obtain pure CuSbS_2_.

## 1. Introduction

**Scheme 1:**
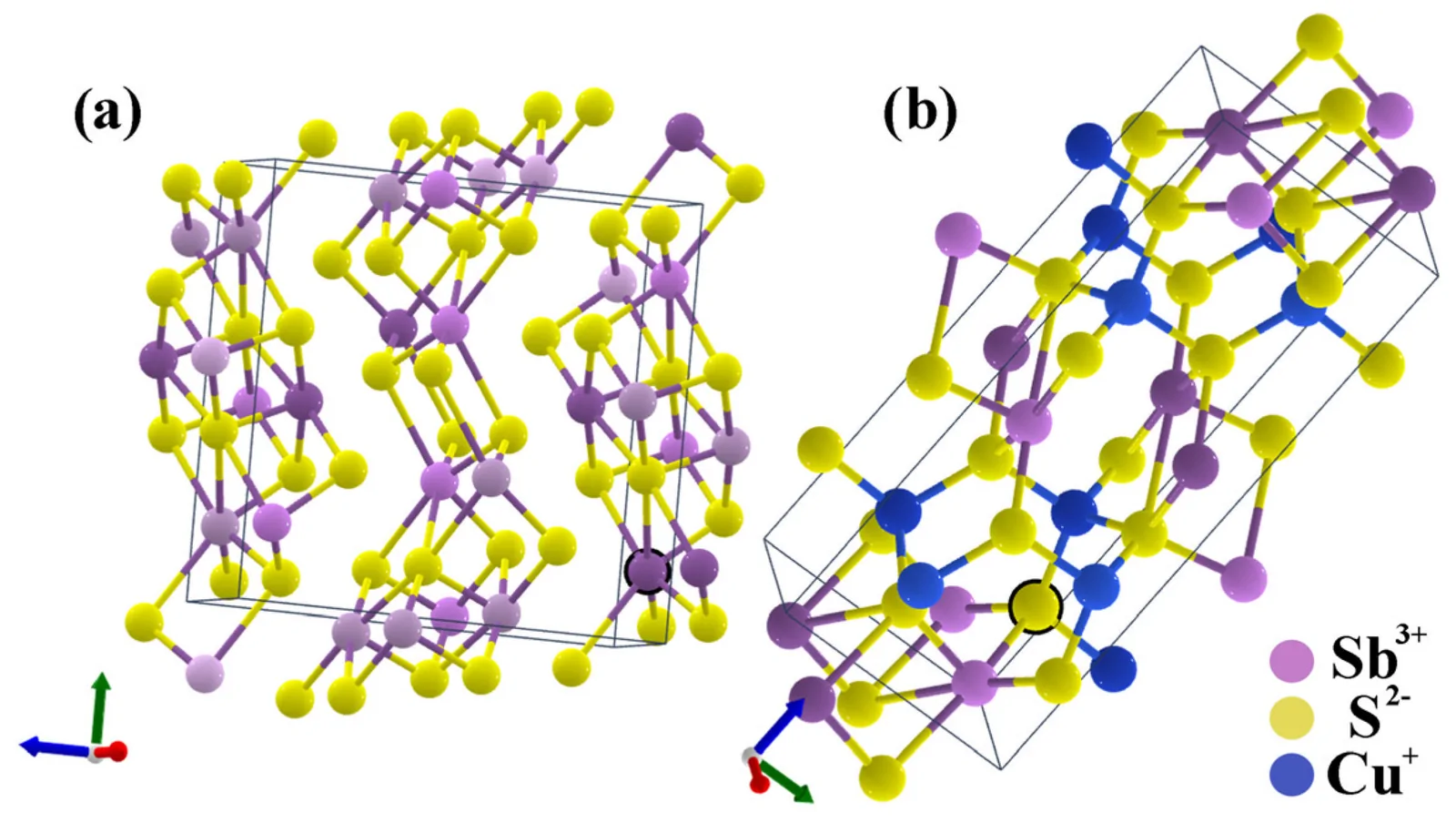
Structures of orthorhombic Pnma: (**a**) Sb_2_S_3_ and (**b**) CuSbS_2_ structures drawn using the open access database The materials project (https://next-gen.materialsproject.org/, accessed on 12 August 2026).

Antimony sulfide, Sb_2_S_3_, is currently an interesting subject of research in the field of photovoltaics owing to its abundant components and suitable optical properties: band gap in the visible-light range (1.5–2.2 eV), high light-absorption coefficient in the range of 10^4^–10^5^ cm^−1^ and a high refractive index (2.5–2.8). For this reason, light-absorber layers based on Sb_2_S_3_ have been recently studied often in photovoltaic devices, especially under low-light conditions [1,2]. Sb_2_S_3_ crystallizes as an orthorhombic structure, with two different Sb positions (coordination numbers 3 and 5) and two S positions (coordination numbers 2 and 3) covalently bonding to create quasi 1D [Sb_4_S_6_]_n_ ribbons interconnected with van der Waals bonds, as schematically represented in Scheme 1a. This structure creates non-isometric electrical and optical properties, which complicate high photoconversion efficiency and application in photovoltaics [3]. Crystalline Sb_2_S_3_ is usually reported to be a direct band gap material with a band gap in the lower range (1.5–1.7 eV), appropriate to be excited by visible light. Amorphous Sb_2_S_3_ has a larger band gap (1.7–2.2 eV), utilizing the narrower spectral range, and has a lower absorption level due to being indirect [4].

The hot-injection synthesis route developed by our group [5,6] enables completely homogeneous mixing with doping ions at any ratio. It also allows fast and uniform nucleation and growth of crystals. Previous studies showed that antimony substitution with copper influences the growth of crystals during synthesis, enhances light absorption and lowers the band gap, so this study was conducted to better understand how copper ions modify the structure, microstructure and optical properties of Sb_2_S_3_ [7,8,9,10].

There is another consequence of the specific structure of Sb_2_S_3_. Structural 1D orientation leads to the formation of 1D microstructures upon the crystallization of particles [3]. It is challenging to deposit these particles uniformly without their spontaneous orientation and agglomeration. Therefore, it is beneficial to identify a method to prevent the one-dimensional growth of particles and to make them as close to spherical as possible before their deposition as a component of a solar cell.

Chalcostibite, CuSbS_2_, is another promising light-absorber material for photovoltaics. It exhibits similar properties to Sb_2_S_3_, including a suitable bandgap (1.4–1.6 eV), large optical absorption coefficient (10^5^ cm^−1^), good stability, nontoxicity and abundant constituents. It crystallizes in an orthorhombic 2D-layered structure (Pnma space group) of Cu-S pentahedra and Sb-S tetrahedra with a unit cell composed of four formula units, as schematically represented in Scheme 1b. Other copper antimony sulfide systems like skinnerite (Cu_3_SbS_4_), fematinite (Cu_3_SbS_4_) and tetrahedrite (C_12_Sb_4_S_13_) exhibit similar optical properties and are worth studying in the field of photovoltaics [11,12,13,14,15,16,17,18,19,20].

Sb_2_S_3_ has an orthorhombic Pnma structure, and it is complicated to predict the radii or preferable position of substituting ions. The nature of bonds is more covalent, and two different coordination numbers (CN) of Sb(III) ions (CN3—dominantly covalent, and CN5—three covalent and two van der Waals bonds) give a complex 1D structure with an unknown radius of Sb [21,22]. Cu(I) typically does not crystallize in this coordination, but if we compare the ionic radii of the two hexacoordinated ions, they are very similar: Sb(III) (0.76 Å) and Cu(I) (0.77 Å); so, a successful substitution may potentially happen [23]. The electronegativities of Cu and Sb are similar, measuring 1.9 and 2.05 on the Pauling scale, so, on that side, they compose a suitable substitution pair (https://pubchem.ncbi.nlm.nih.gov/ptable/electronegativity/ (accessed on 1 July 2026)).

## 2. Results and Discussion

### 2.1. XRD Analysis

**Figure 1:**
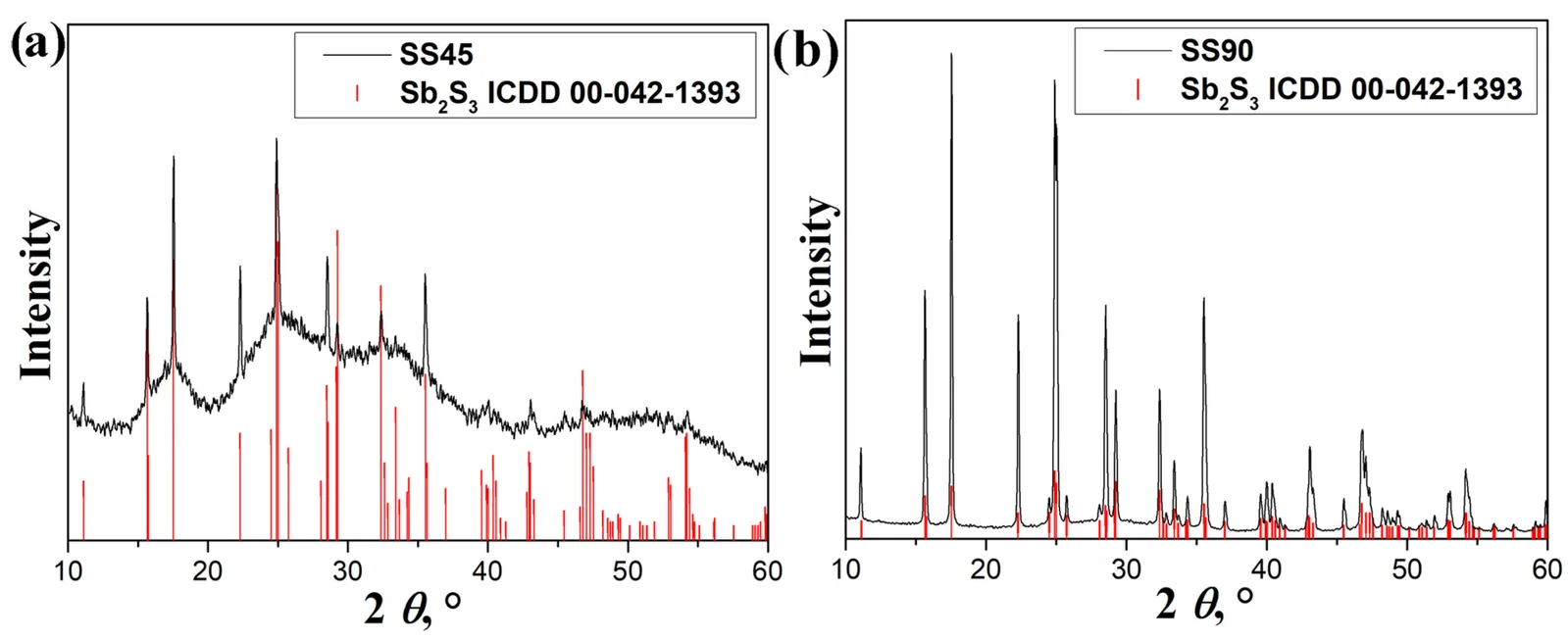
XRD diffraction patterns of (**a**) SS45 and (**b**) SS90 powders.

The development of the structure during hot injection in the initial period is presented in Figure 1 at the angles of the most prominent peaks. Orthorhombic Sb_2_S_3_ is the first phase that crystallizes from the amorphous phase, and it is already evident after 45 s, with better development and growth of crystals after 90 s.

**Figure 2:**
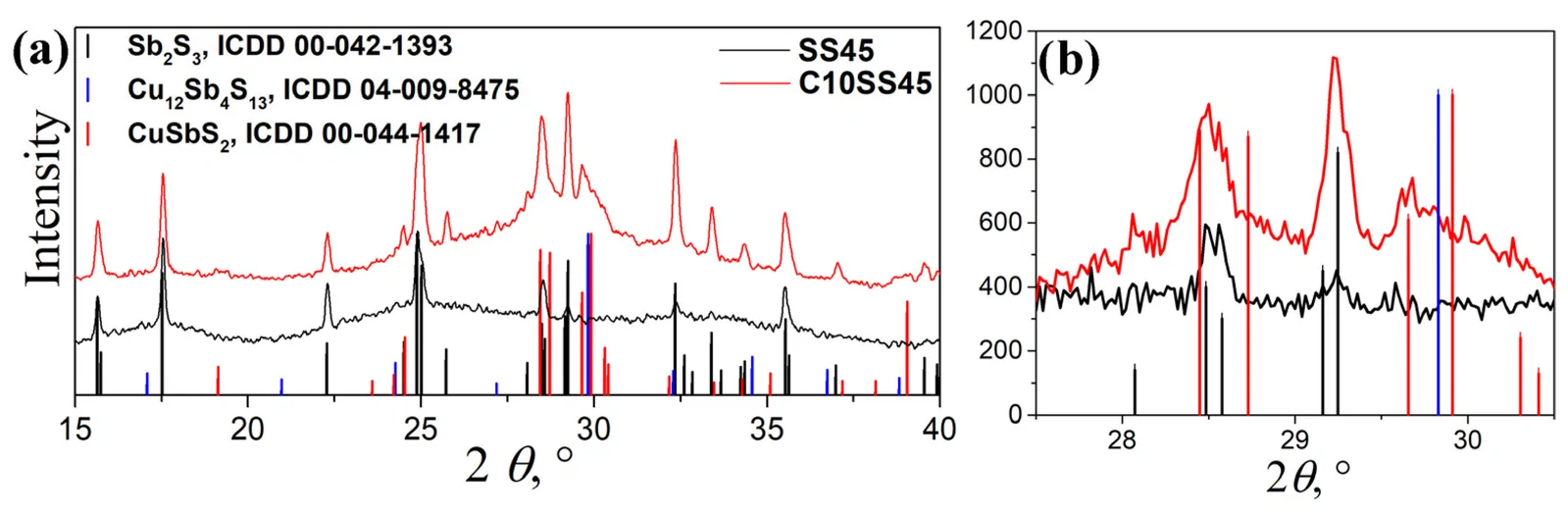
XRD patterns of SS45 and C10SS45 (**a**) and enlarged part of the patterns for 2*θ* between 27.5 and 30.5° (**b**).

In the presence of Cu substituting 10% of Sb, the crystallinity mildly improves after 45 s of synthesis, as can be seen in Figure 2a. A slight orientation change is visible in the enlarged spectra at 2*θ* = 27.5–30.5° in Figure 2b. Copper-containing phases were not detected.

**Figure 3:**
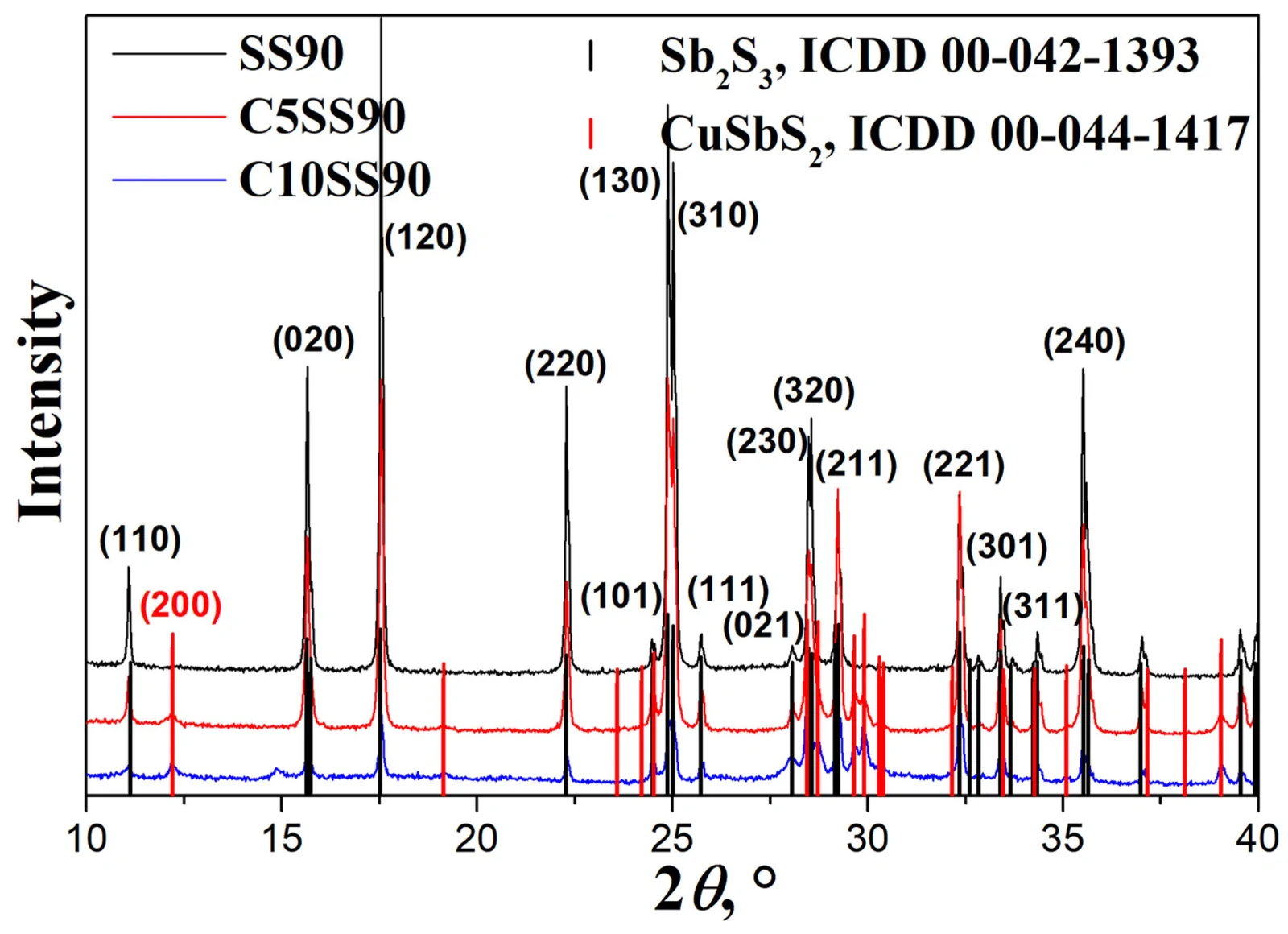
XRD patterns of SS90, C5SS90 and C10SS90 powders.

**Figure 4:**
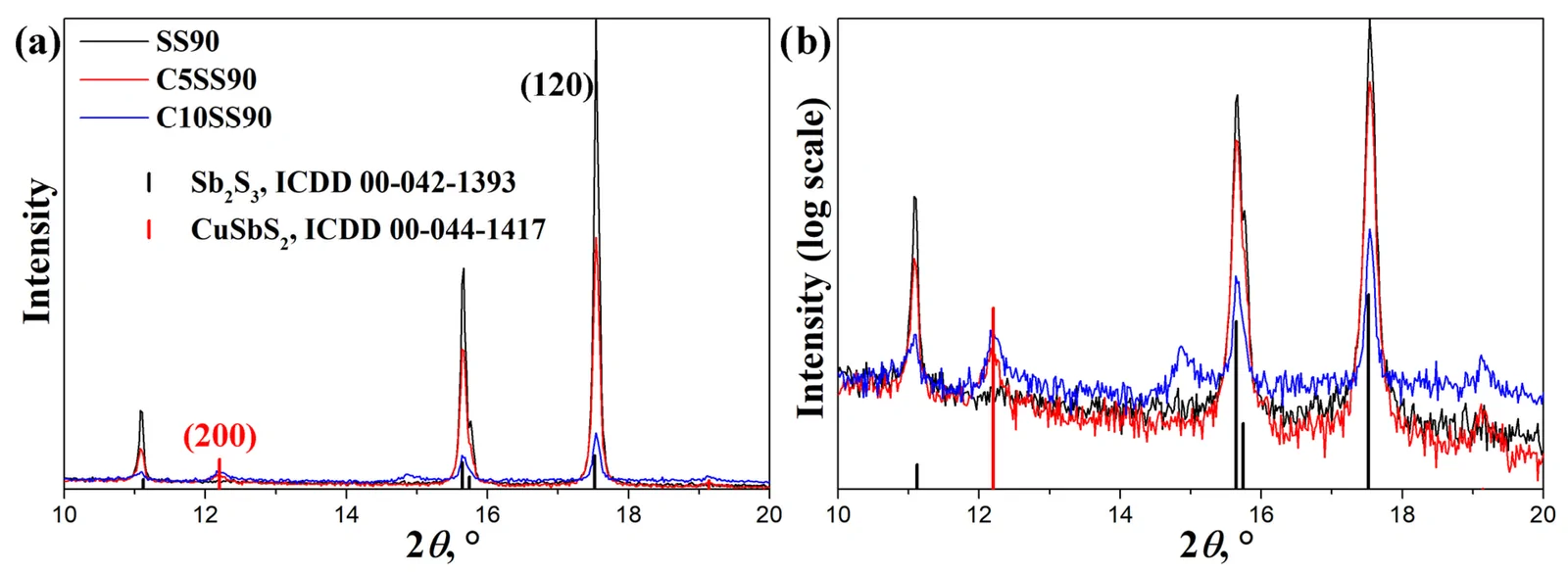
Enlarged regions of interest of XRD patterns of SS90, C5SS90 and C10SS90 powders with linear (**a**) and logarithmic (**b**) intensity scales.

After doubling the reaction time to 90 s, Cu-substituted Sb_2_S_3_ samples show lower crystallinity than pristine ones, as can be seen in Figure 3, with a clearly modified orientation, and the most intense peaks for C10SS90 are (211) and (221). In SS90, (120), (130), (230), (220) and (240) planes had the maximal intensity, as well as for C5SS90, though a decrease in these peaks and increase in (211) and (221) can be observed for the 5% Cu substitution level. The position of the peaks is unchanged, implying the copper ions may not actually be incorporated into the structure of Sb_2_S_3_, but rather some other phase. The impact of Cu substitution on the structure is more obvious at the enlarged section of 2*θ* between 10 and 20°, presented in Figure 4a. The highlighted low-intensity peak of the CuSbS_2_ phase is obvious if the intensity in this region is presented with a logarithmic scale (Figure 4b). A significant secondary CuSbS_2_ phase formed at both the 5% and 10% substitution levels, which is often reported for Cu-substituted Sb_2_S_3_ [24]. The content of phases, calculated based on the (120) peak of Sb_2_S_3_ and (200) peak of CuSbS_2_, shows a CuSbS_2_ phase composed 6.6% wt of C5SS sample and 27.0% wt of C10SS sample, which corresponds to 5.0% and 16.7% of Cu incorporation into the product, in the form of the CuSbS_2_ phase. All the other secondary phases remained under the limit of detection. The reaction yield is not 100% and that may be the reason for more Cu in the product than in the added reactants.

The chemistry of synthesis solutions strongly influences the kinetics of processes. When Sb(III) acetate was used as a source of Sb instead of SbCl_3_, the powders remained completely amorphous after 90 s, and a longer reaction time was needed to achieve crystallization (Figure S1 in the Supplementary Materials). The presence of copper did not significantly influence crystallinity, similarly to the synthesis with chlorides present. Chlorides in the system promote precipitation.

### 2.2. Raman Analysis

**Figure 5:**
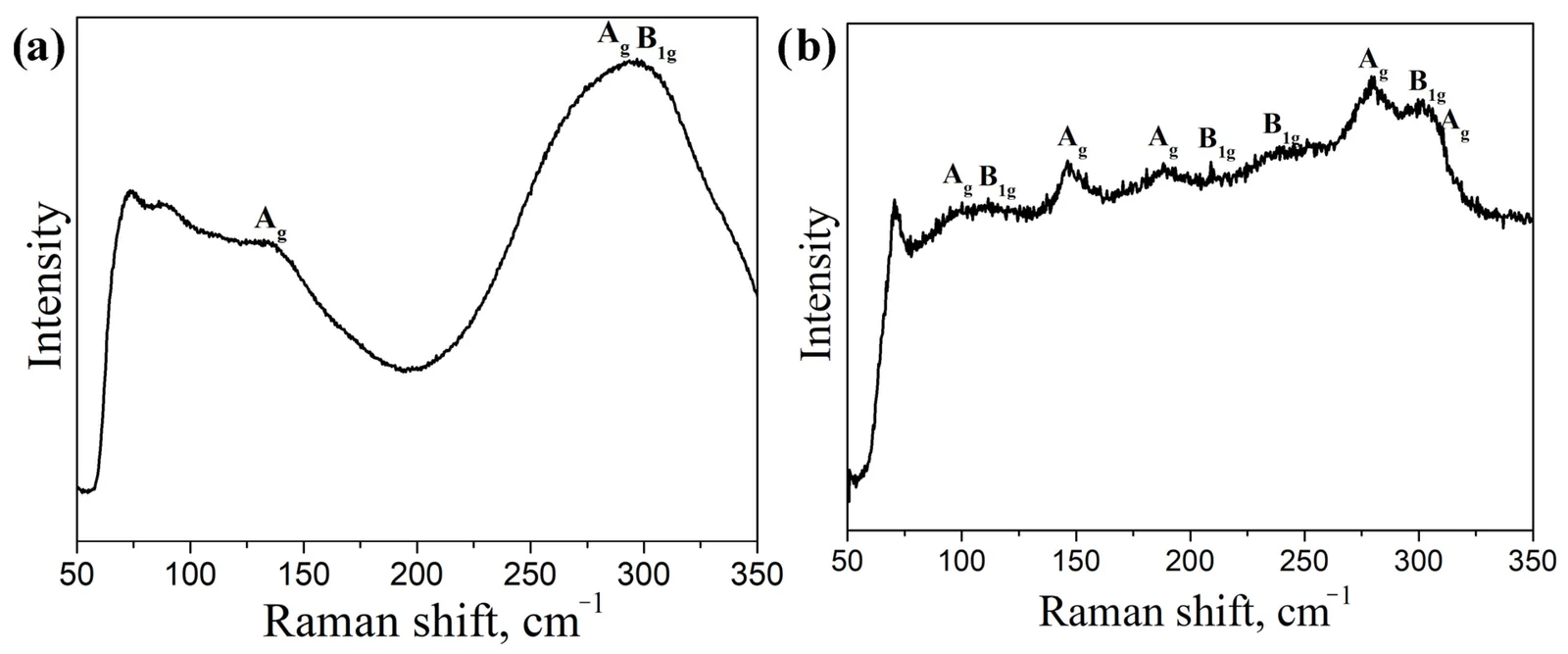
Raman spectra of (**a**) SS45; (**b**) SS90.

The amorphous SS45 powder (Figure 5a) shows the characteristic most-intense A_g_ modes of Sb-S bonds, which are starting to form [25,26]. Crystalline SS90 samples exhibit bright rod-like particles and gray powdery areas under optical microscope (Figure S2a,b), but those two regions show very similar Raman responses (Figure S2d) with nine of the typical 10 A_g_ and B_1g_ modes of Sb_2_S_3_ crystalline phase observed, confirming the similar structure between the two morphologies [27]. Bigger white particles observed in small number in powders (Figure S2c) were identified by the Raman analysis of spectra presented in Figure S2e as the bulk antimony phase (bands at 109 and 146 cm^−1^), though antimony is present in some other regions independently of the observed microstructures. Peaks related to the Sb_2_O_3_ oxide phase may become evident at 188 and 251 cm^−1^, like it may be seen in Figure S2e, but the oxide phase is not inherently present in powders. When using the lower power of the laser (neutral density filter ND5 instead of ND3 or ND1) in the Raman analysis (like in Figure 5b), the oxides are absent, so their occurrence is related to thermal oxidation under the laser beam in an air atmosphere [26,27].

**Figure 6:**
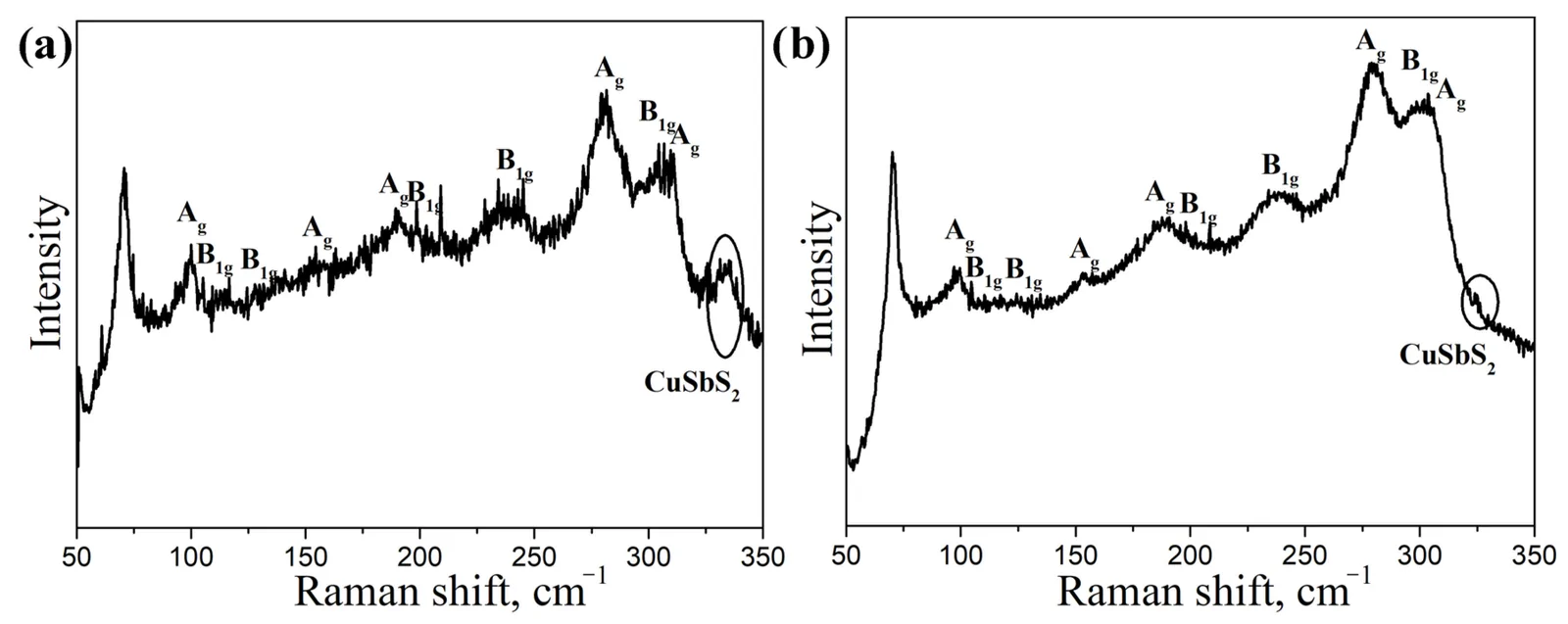
Raman spectra of (**a**) C5SS90; (**b**) C10SS90.

Cu-doped samples (Figure 6) did not exhibit many differences in their Raman responses except for the noticeable presence of a band at around 330 cm^−1^, corresponding to the CuSbS_2_ phase in Cu-substituted samples, also detected by XRD analysis [13].

### 2.3. TEM Analysis

**Figure 7:**
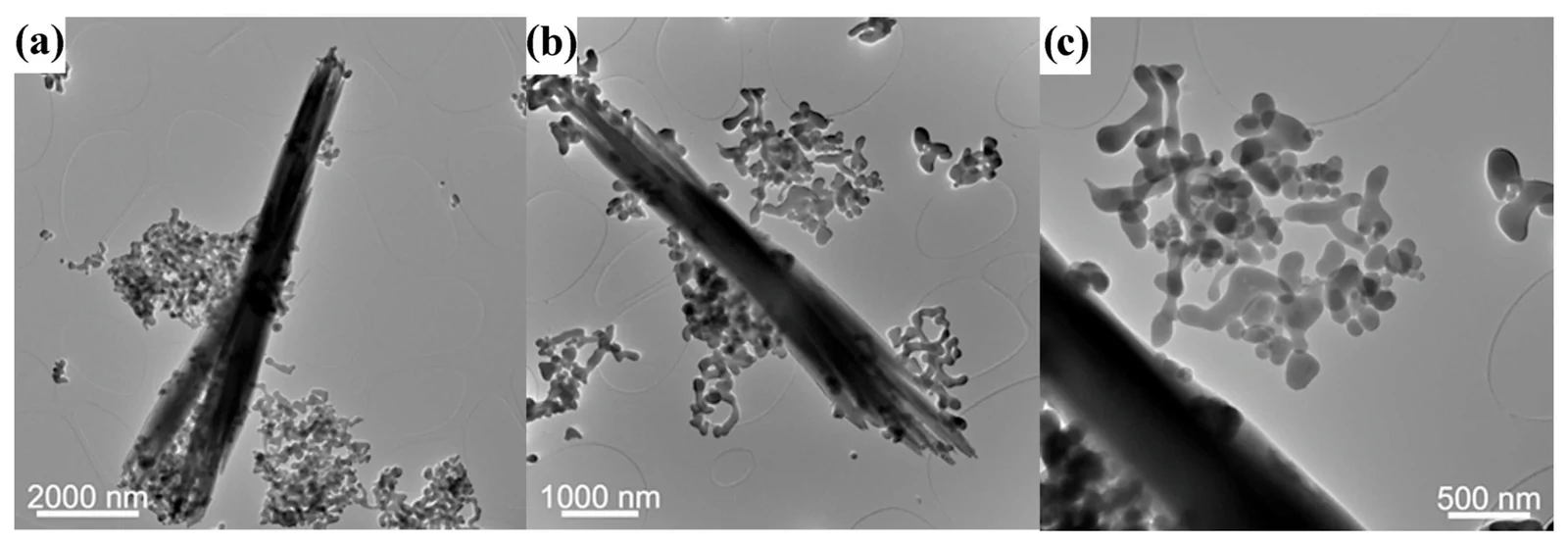
TEM images of SS45 powder at (**a**) 7000×, (**b**) 10,000×, and (**c**) 22,000× magnifications.

**Figure 8:**
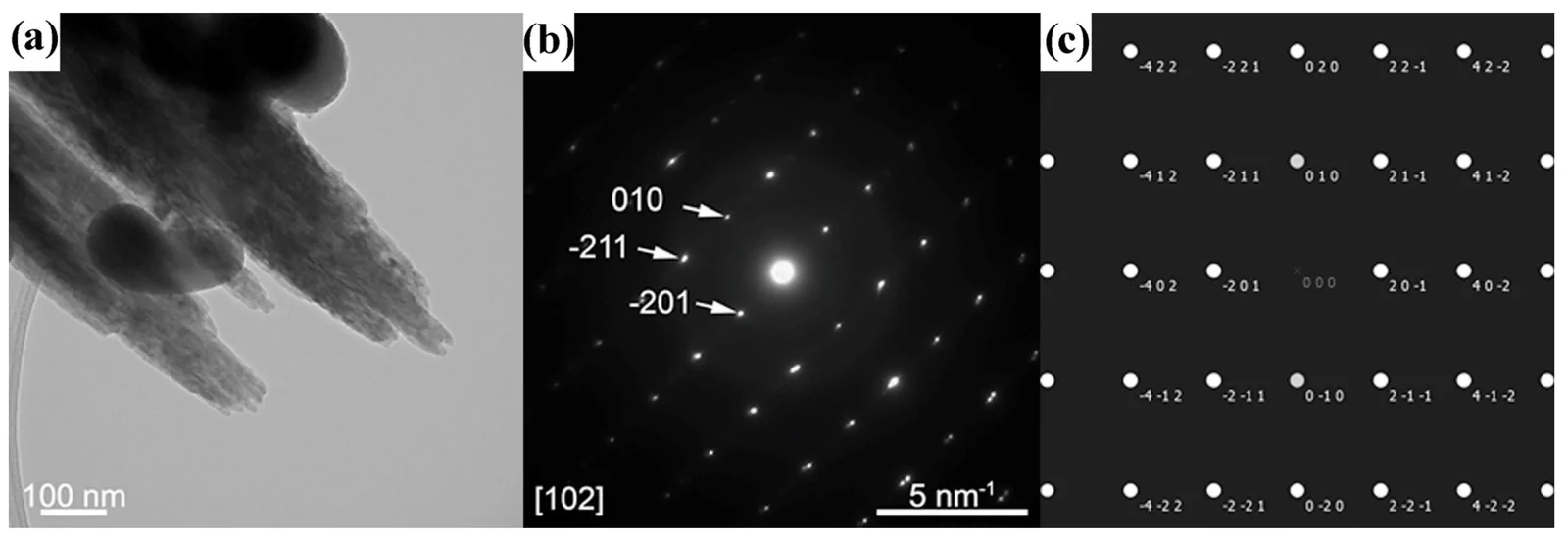
(**a**) TEM images of the end of the Sb_2_S_3_ microrod from the SS45 sample, (**b**) selected area diffraction pattern and (**c**) simulated diffraction pattern oriented along the [102] zone axis.

Sample SS45 exhibited several-microns-long needle-like particles bundled together, as well as smaller particles with irregular shapes, ranging from spherical particles with a ~100 nm diameter to elongated structures made of several connected spherical particles, as seen in Figure 7. The selected area electron diffraction (SAED) pattern of the crystalline microrods observed in Figure 8a confirms that the crystalline phase is Sb_2_S_3_ as seen in Figure 8b. The spots that correspond to the lattice planes (010)—3.83 Å, (−211)—3.05 Å and (−201)—5.05 Å are marked. The zone axis [102] is perpendicular to these planes, giving the crystal orientation. The same results were obtained by measuring the interplanar distances in the spherical particle in the HRTEM image using Fourier transform (FFT) in Figure S3 (Supplementary Materials). Both the SAED pattern and the FFT are comparable with a simulated diffraction pattern image, as seen in Figure 8c, confirming the Sb_2_S_3_ phase.

**Figure 9:**
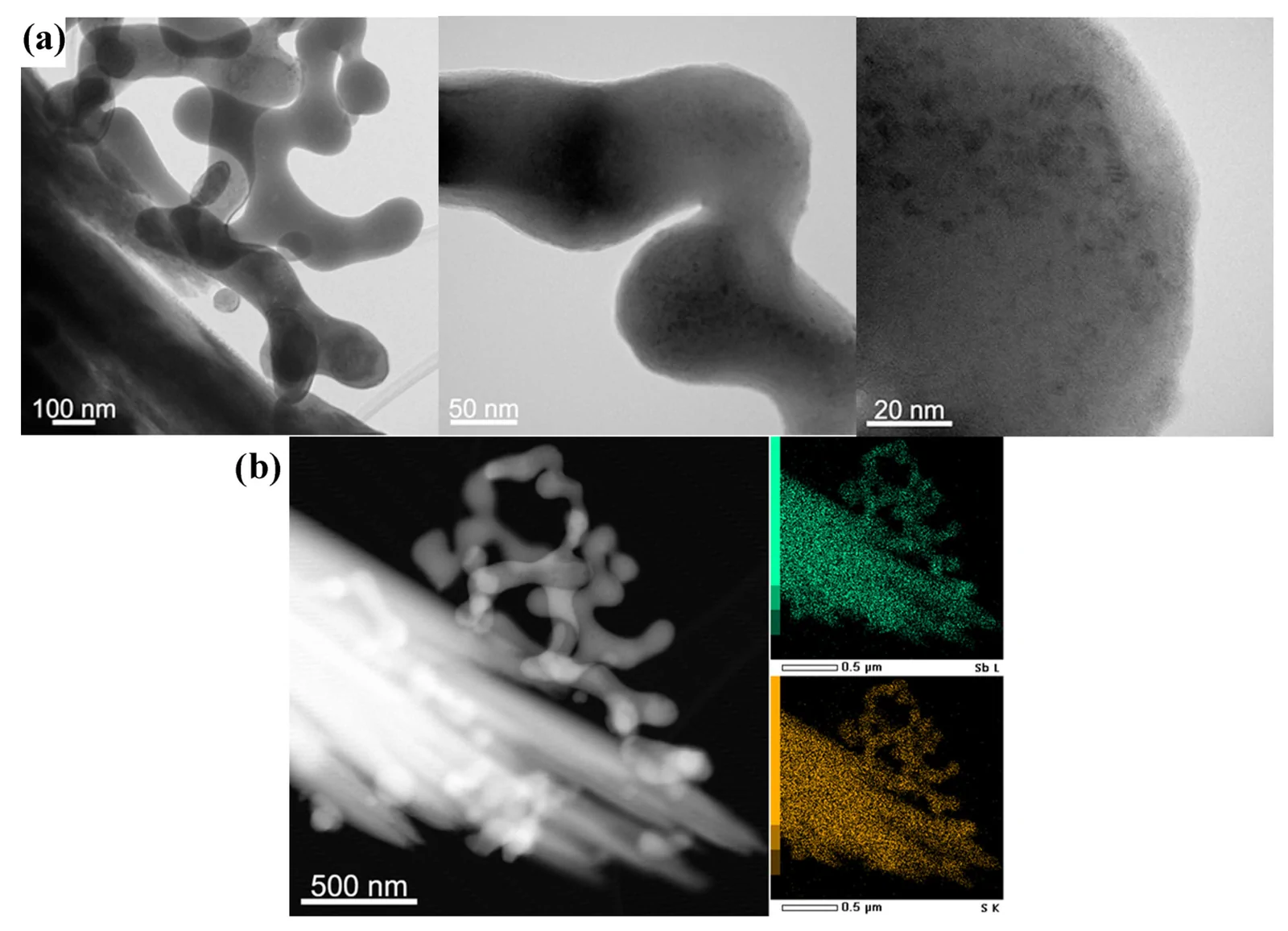
TEM images of the conjoined spherical nanoparticles in the SS45 sample (**a**); STEM image and EDX elemental analysis showing the Sb and S distribution (**b**).

The images in Figure 9a reveal that the spherical conjoined nanoparticles differ structurally. They are mostly amorphous, but a crystalline phase is also observed in them. The crystalline phase was confirmed to be Sb_2_S_3_. The mapping (Figure 9b) and EDS spectra (Figure S4) show uniform distributions of Sb and S in all particles. HRTEM images revealed that these nanoparticles in SS45 may be both amorphous and crystalline, showing that the characteristic process of the 1D rod-like particle formation of Sb_2_S_3_ takes place 45 s into the reaction. According to XRD, this sample is predominantly amorphous with initial crystallization observed by the separation of the sharp low-intensity Sb_2_S_3_ peak (Figure 1 and Figure 2), meaning the amorphous nanoparticle aggregates are the dominant morphology.

**Figure 10:**
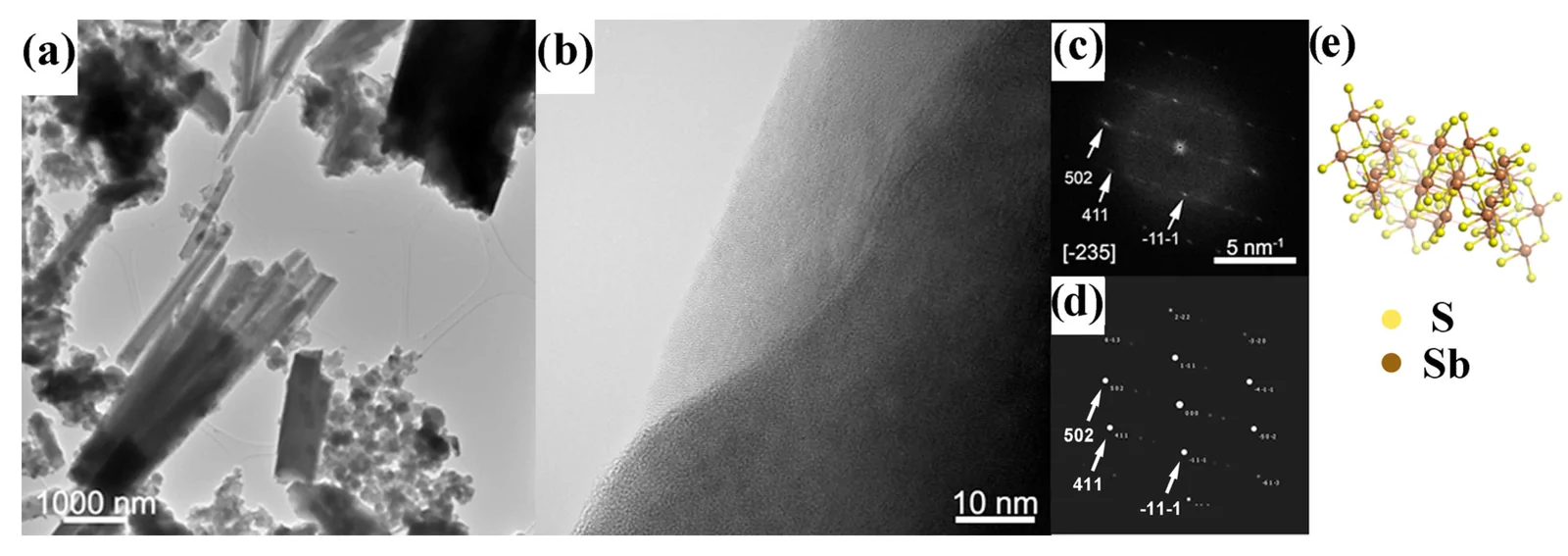
(**a**) Low-magnification TEM images of the SS90 sample, (**b**) HRTEM image of the elongated particle, (**c**) FFT pattern corresponding to it, (**d**) simulated diffraction pattern of a Sb_2_S_3_ crystal oriented along the [−235] zone axis and (**e**) structural model of the Sb_2_S_3_ crystal oriented as in the HRTEM image.

The TEM investigation of sample SS90 reveals a similar presence of two distinct morphologies (Figure 10). Elongated crystalline rod-like particles are present to greater extent, alongside a significant amount of particles with different shapes. The elongated particles are of similar sizes to those in SS45 powders with a length above 1000 nm (reaching up to 5000 nm) and width between 200 and 300 nm (reaching over 1000 nm), with an aspect ratio of 5:1. The HRTEM image in Figure 10b was analyzed by taking the Fourier transform of the area in which lattice fringes were visible. The obtained FFT pattern (Figure 10c) displays bright spots compatible with the (502), (411) and (−11−1) interplanar distances of Sb_2_S_3_. The simulated diffraction pattern and structural model of the Sb_2_S_3_ crystal are displayed in Figure 10d,e.

**Figure 11:**
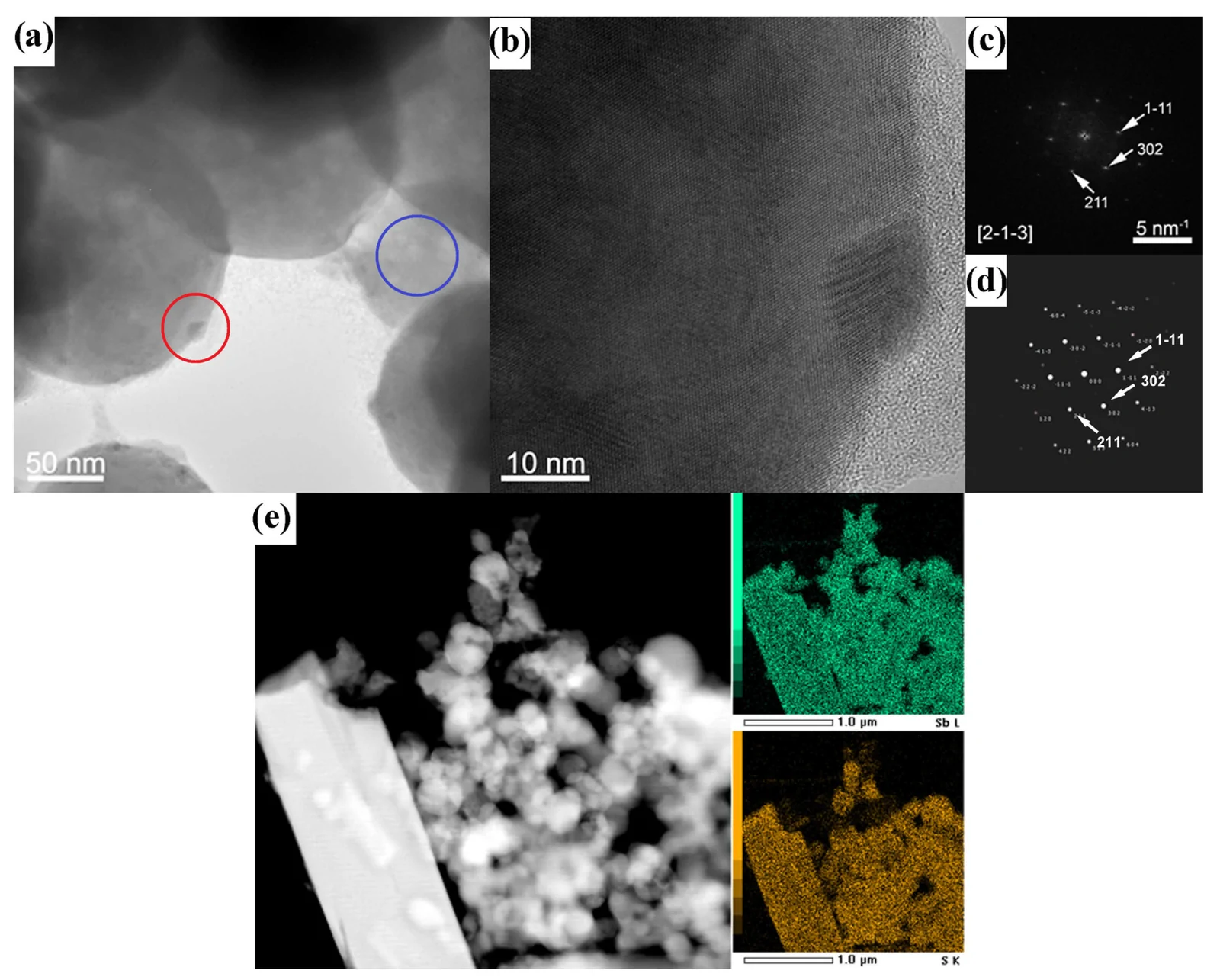
(**a**) TEM images of the spherical particle region of the SS90 nanopowder, with indicated nanocrystalline (red) and porous-like (blue) features; (**b**) HRTEM image in the red circled area; (**c**) FFT pattern of the HRTEM image; (**d**) simulated diffraction pattern of the Sb_2_S_3_ crystal oriented along the [2−1−3] crystallographic direction and (**e**) STEM dark-field image of the SS90 power, with elemental distribution maps of Sb and S.

The spherical nanoparticles presented in TEMs in Figure 11a are mostly above 100 nm in diameter and appear amorphous, but small nanocrystalline domains may be observed on their surfaces—like the red circled area in Figure 11a. Some particles display porous-like features, as those seen in the blue circle in Figure 11b. The FFT analysis performed on such spherical nanoparticles, presented in Figure 11c, indicates that the lattice fringes visible in the HRTEM image correspond to those of a Sb_2_S_3_ crystal oriented along the [2−1−3] zone axis. The comparison with the simulated electron diffraction pattern (Figure 11d) confirms that the phase is Sb_2_S_3_. The phase of the nanoparticles present on the surface of the large spherical particles was not identified.

The STEM-EDX analysis performed on the area in Figure 11e shows that there are some areas with amorphous particles in which the S signal is not present, which may explain the Sb-rich areas observed under Raman analysis (Figure 5b). The rest of the map displays homogeneous distributions of S and Sb in both spherical and elongated nanoparticles, reaffirming that the two types of particles belong to the same phase but appear in different morphologies. No differences in the concentration were observed. Small spots can be seen in some spherical particles with a different level of contrast to the surrounding material, resembling porous areas in Figure 11a, which is usually associated with the smaller average atomic number of the material in that area or indicates that there is a pore in the crystal structure [28].

**Figure 12:**
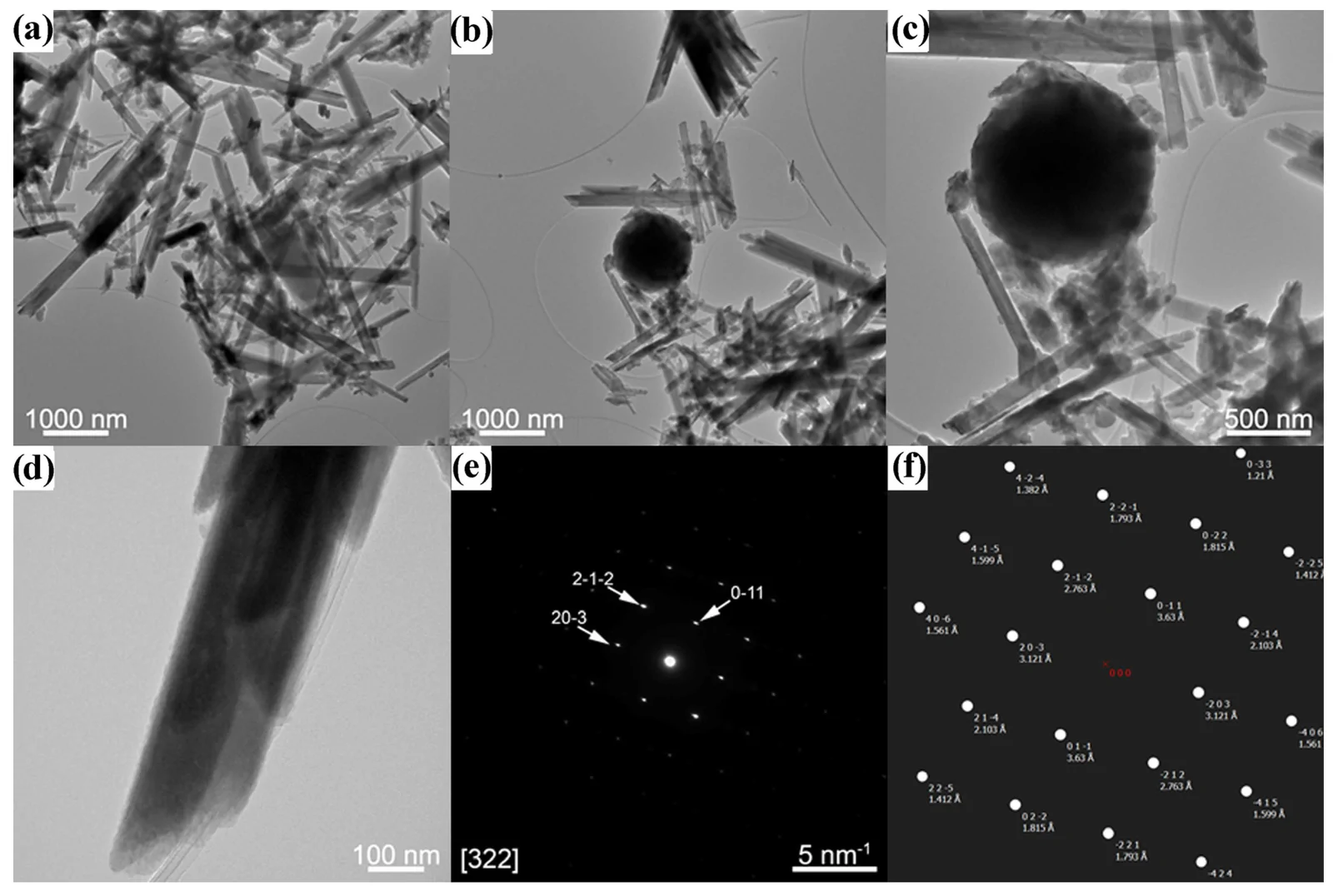
(**a**–**d**) TEM images of the C5SS90 sample at different magnifications with (**e**) selected area diffraction patterns of regions observed at (**d**), and (**f**) simulated diffraction pattern of the Sb_2_S_3_ crystal oriented along the [322] zone axis.

Three types of morphologies were identified by TEM analysis of the C5SS90 sample (Figure 12). The dominant one consists of large elongated particles whose lengths surpass 1000 nm with thicknesses below 200 nm. They have a larger length-to-thickness ratio than similar particles in the unsubstituted sample. The second type of particles are irregularly shaped nanoparticles with smaller dimensions (below 100 nm), agglomerated along the edges of elongated particles, and this morphology exists only in the presence of Cu. The overall content of elongated particles has increased. There is also a low amount of spherical nanoparticles that seem to be amorphous, which are mostly 100 nm in diameter. Spherical particles larger than 500 nm can be seen (Figure 12b,c), and the STEM mapping in Figure S5 shows them to be in the antimony-rich phase, explaining the bulk antimony observed by Raman analysis in some areas (Figure 5). EDX analysis (Figure S7) revealed significant traces of Cl in this region, but not in areas with elongated particles.

The SAED pattern of the elongated particle (Figure 12e) confirms its monocrystalline structure. The spots corresponding to the following lattice planes were measured: (20−3)—3.12 Å, (2−1−2)—2.76 Å and (0−11)—3.63 Å (these are indeed the (230), (221) and (101) planes indicated in Figure 3, only with different orders of the plane annotation in structure cards used in TEM analysis). The orientation of the Sb_2_S_3_ crystal in which these planes are visible simultaneously is in the [322] zone axis (Figure 12f).

**Figure 13:**
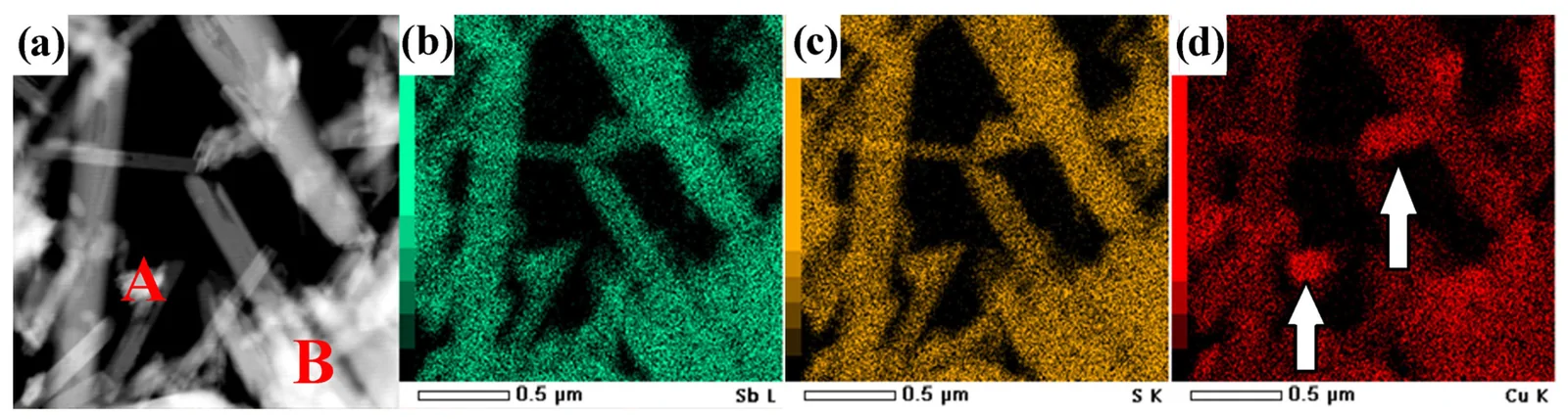
STEM mapping of the C5SS90 powder: the selected area microstructure with marked A and B characteristic regions (**a**), and mappings of Sb (**b**), S (**c**) and Cu (**d**) (arrows are indicating the Cu-rich regions).

**Figure 14:**
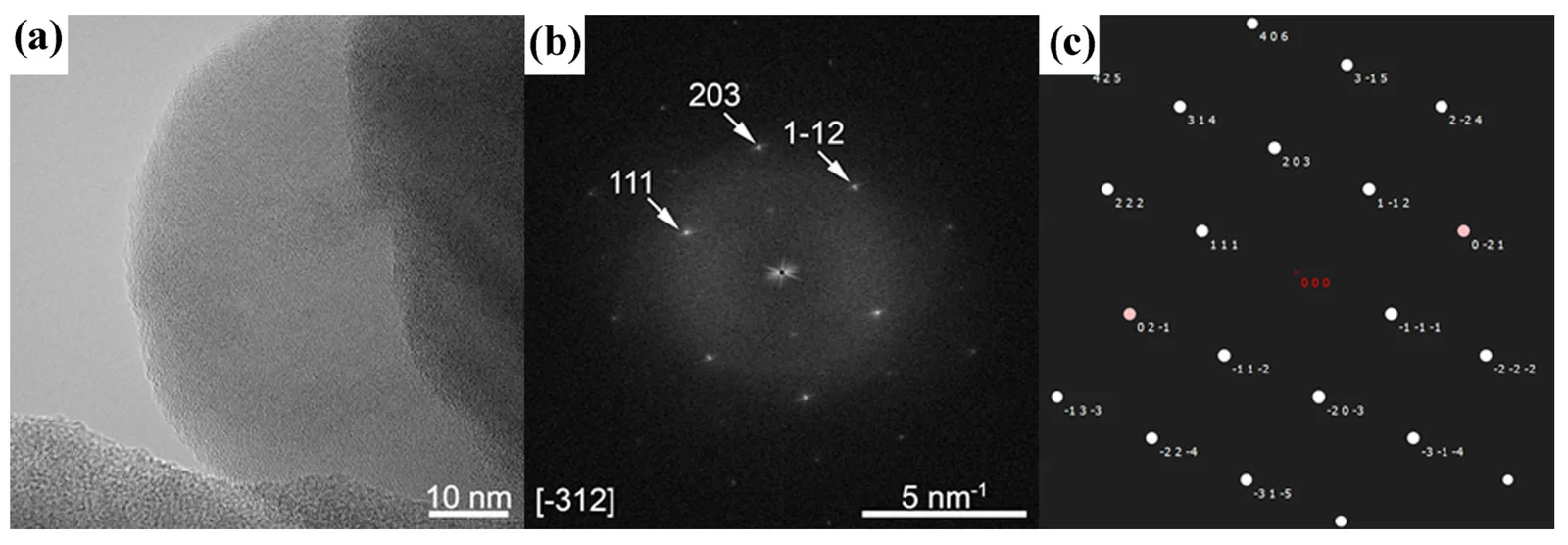
(**a**) HRTEM images of a well-oriented particle from the Cu-rich agglomerate, (**b**) FFT images corresponding to it and (**c**) simulated diffraction pattern of CuSbS_2_ crystal oriented along the [−312] zone axis.

Copper was present in all particles at a similar level, and more than expected based on its doping level (according to EDX analysis presented in Figure S6). It is distributed quite uniformly, but it is possible to notice in the STEM mapping in Figure 13 that there are small regions where it is present in Cu-rich agglomerates (marked with arrows in Figure 13d), but along with Sb and S. The Cu-rich agglomerates are the smaller crystalline platelet-like particles, described as a second morphology. EDX analysis shows they exhibit roughly three times more Cu and somewhat less Sb and S than other particles (Figure S7), implying the presence of a Cu-rich secondary phase and an almost-matching CuSbS_2_ elemental composition. A well-oriented particle from a Cu-rich agglomerate (Figure 14) was determined to have an interplanar distance corresponding to the following lattice planes of the CuSbS_2_ crystal: (111) of 3.13 Å, (203) of 2.55 Å and (1−12) of 2.94 Å. The orientation that exhibits these planes is [−312] and the FFT measurement is confirmed by the comparison with the simulated electron diffraction pattern of the CuSbS_2_ crystal with the same orientation (Figure 14c).

**Figure 15:**
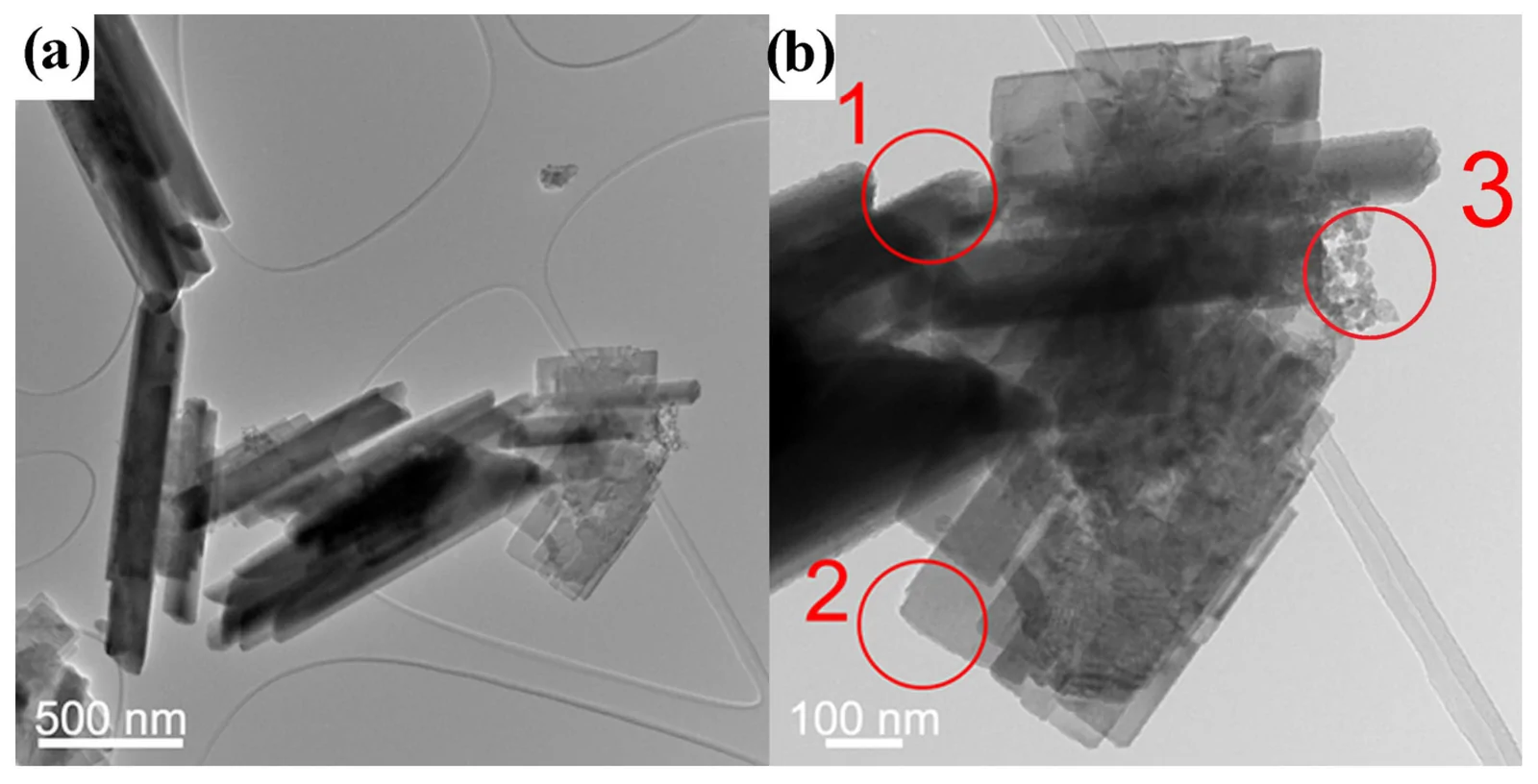
TEM images of C10SS90 powder (**a**), with enlarged section with marked morphologies of rod particles (1), platelets (2) and spherical nanoparticles (3) (**b**).

The TEM investigation of C10SS90 powders presented in Figure 15 revealed at least three morphologies. The most abundant are elongated particles (1000 nm × 200 nm), typical for Sb_2_S_3_, marked with 1 in Figure 15b, but there are also thicker rods present. The other main morphology (marked with 2 in Figure 15b) can be described as an almost rectangular sheet that has low thickness, which gives it a light contrast. Similar sheets can be seen to clearly overlap, a fact that suggests that its thickness along the view-axis is small. The other two dimensions are quite similar in size, reaching several hundred nanometers.

**Figure 16:**
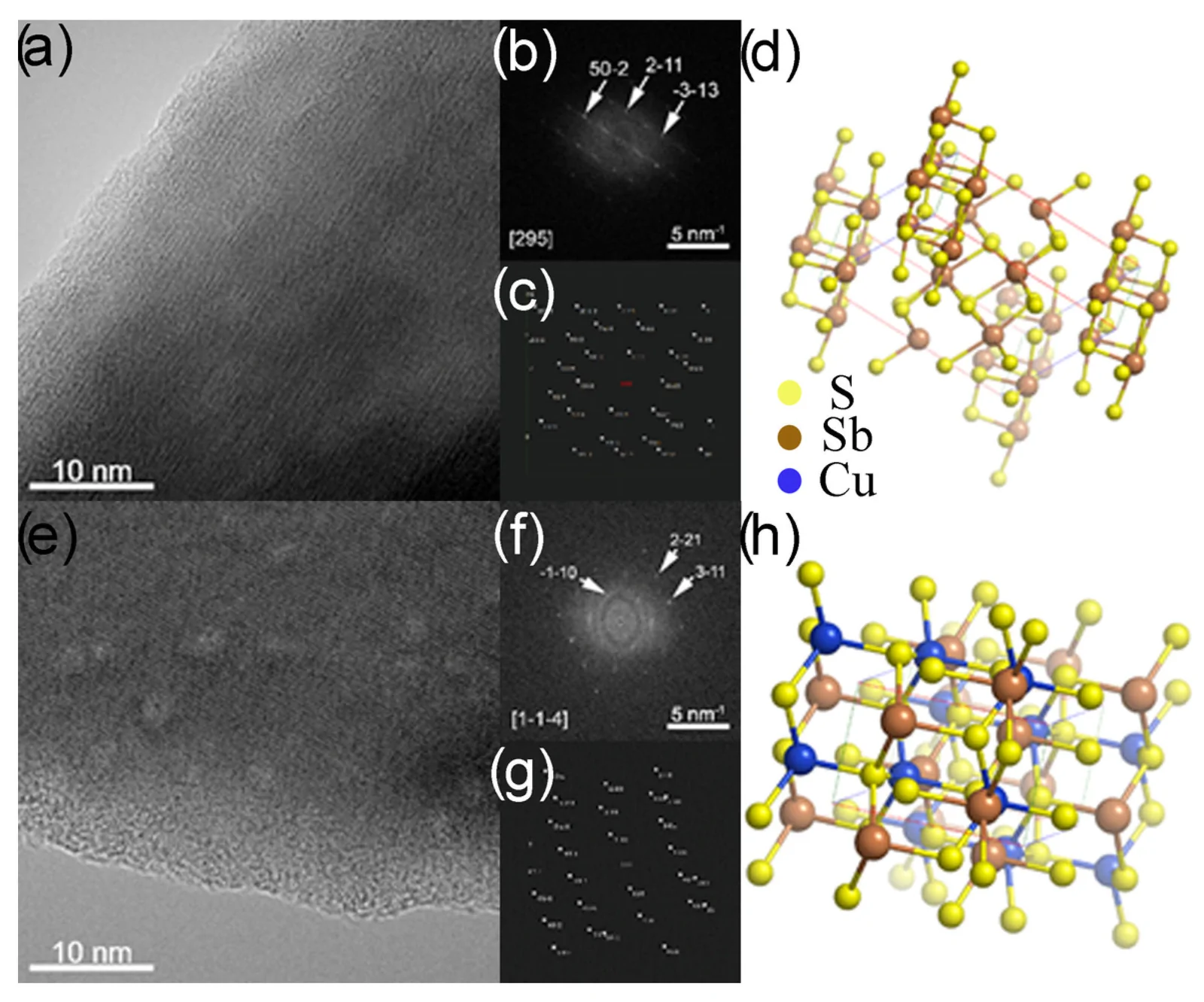
HRTEM images of the elongated particle (**a**) and sheet particle (**e**) of C10SS90 powder; Fourier transform (**b**,**f**); simulated diffraction pattern of Sb_2_S_3_ along the [295] zone axis (**c**); structural model of Sb_2_S_3_ oriented along the [295] axis (**d**); simulated diffraction pattern of CuSbS_2_ along the [1−1−4] zone axis (**g**) and structural model of CuSbS_2_ along the [1−1−4] zone axis (**h**).

The HRTEM investigation of the two types of particles reveals that type 1 can be identified as Sb_2_S_3_, with its HRTEM image presented in Figure 16a. The Fourier transform of the image was performed (Figure 16b) and the spots were measured to correspond to the Sb_2_S_3_ lattice interplanar distances. The orientation of the Sb_2_S_3_ crystal in the image was deduced to be along the [295] zone axis, meaning it has a [295] Bravais lattice vector parallel to the electron beam. The comparison with the simulated diffraction pattern of a single crystal oriented along the same direction (Figure 16c) confirms that the phase is Sb_2_S_3_, with the structure represented in Figure 16d.

The analysis of type 2 sheets on HRTEM (Figure 16e) and the FFT pattern (Figure 16f) correspond to the interplanar distance of the CuSbS_2_ phase. The FFT pattern is compatible with the simulated diffraction pattern of a single-crystal CuSbS_2_ phase oriented along the [1−1−4] zone axis (Figure 16g), thus indicating that the type-two sheets are composed of CuSbS_2_, with the structural model shown in Figure 16h.

Type 3 morphologies are spherical nanoparticles observed in all the samples alongside the long crystalline rods and marked by area 3 in Figure 15b and Figure S8. These nanoparticles were scarcer in this sample and mostly amorphous with some crystallized areas (whose structure could not be determined). The diameter of spherical particles varies a lot, going from 10 to 100 nm.

**Figure 17:**
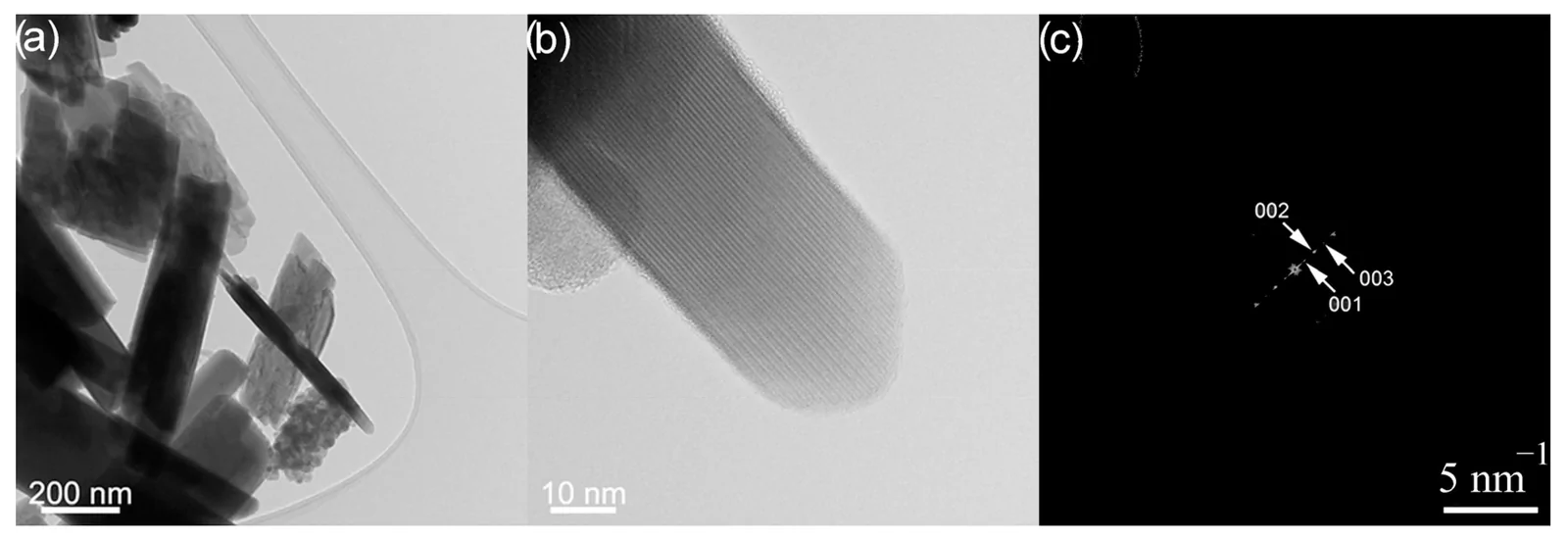
(**a**) TEM images and (**b**) HRTEM images of type 4 particles of C10SS90 powder with (**c**) FFT pattern corresponding to image (**b**) in which the interplanar distances belonging to the CuSbS_2_ phase were measured.

Another much rarer morphological type (type 4) can be seen in Figure 17. It has an elongated, cylindrical shape with a length much larger than the width. The length has a dimension in the range of hundreds of nanometers while the width does not surpass 100 nm. This shape is similar to the rod-like particles of Sb_2_S_3_, though they are smaller and have a somewhat larger aspect ratio. The FFT analysis indicates that this particle has the structure of CuSbS_2_.

**Figure 18:**
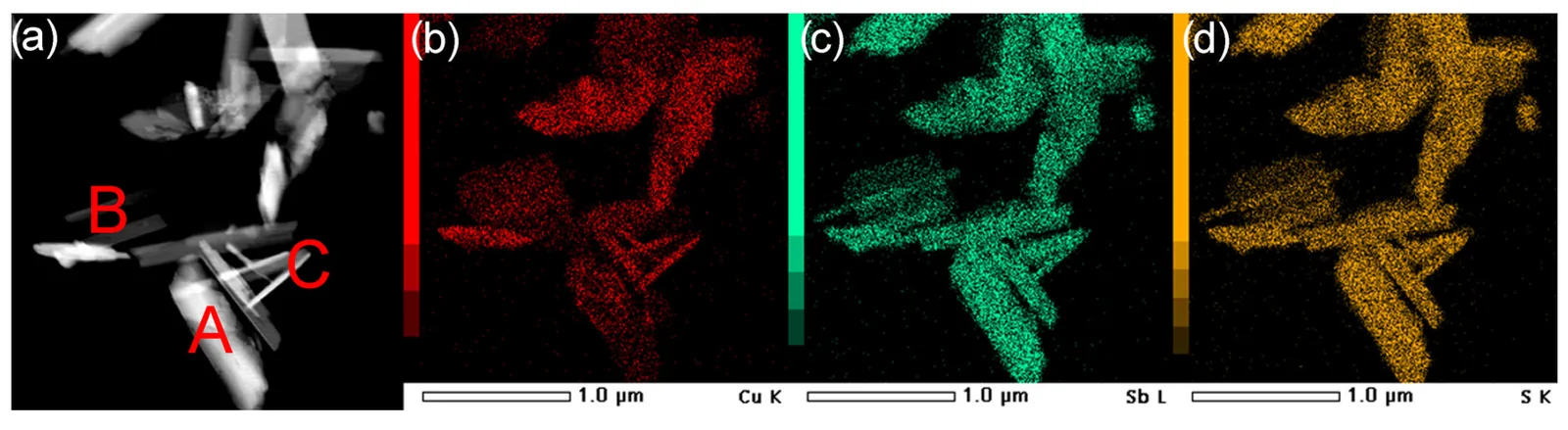
STEM EDX mapping of C10SS90 powder: (**a**) mapped area with indicated characteristic A, B and C regions; (**b**) Cu, (**c**) Sb and (**d**) S mapping.

STEM EDX mapping was performed on an area that contained all described crystalline morphologies (Figure 18). The STEM image and the elemental maps corresponding to Cu, Sb and S are shown, confirming the almost completely uniform distribution of all three elements, with copper absent in some parts of the rod-like particles. EDX spectra of selected regions are presented in Figure S9. A type 1 elongated particle is represented in region A and its chemical composition shows that a small quantity of Cu (4.4 at. %) is present. The type 2 sheet particle in region B coincides with the composition of CuSbS_2_. Type 4 cylindrical CuSbS_2_ nanoparticles in region C had a somewhat smaller Cu/Sb ratio than the type 2 morphology, probably because the analyzed area was small and it overlapped with the Sb_2_S_3_ phase in the surrounding areas.

**Figure 19:**
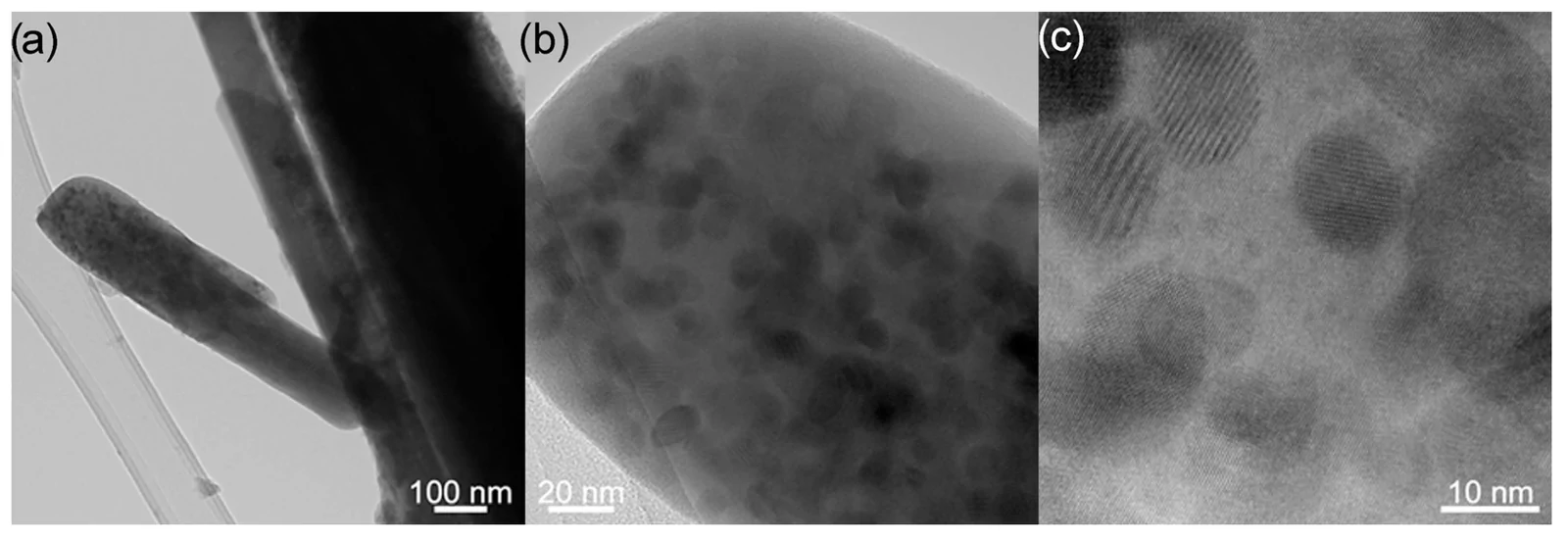
Lower-magnification TEM images of C10SS90 powder (**a**) and TEM images of the same area at higher magnifications (**b**,**c**).

On some of the type 4 cylindrical CuSbS_2_ particles, interesting nanoparticulate regions were observed, as indicated in Figure 19. Numerous quasi-spherical, unidentified nanoparticles with diameters of around 10 nm are densely distributed over their surface.

### 2.4. XPS Analysis

**Figure 20:**
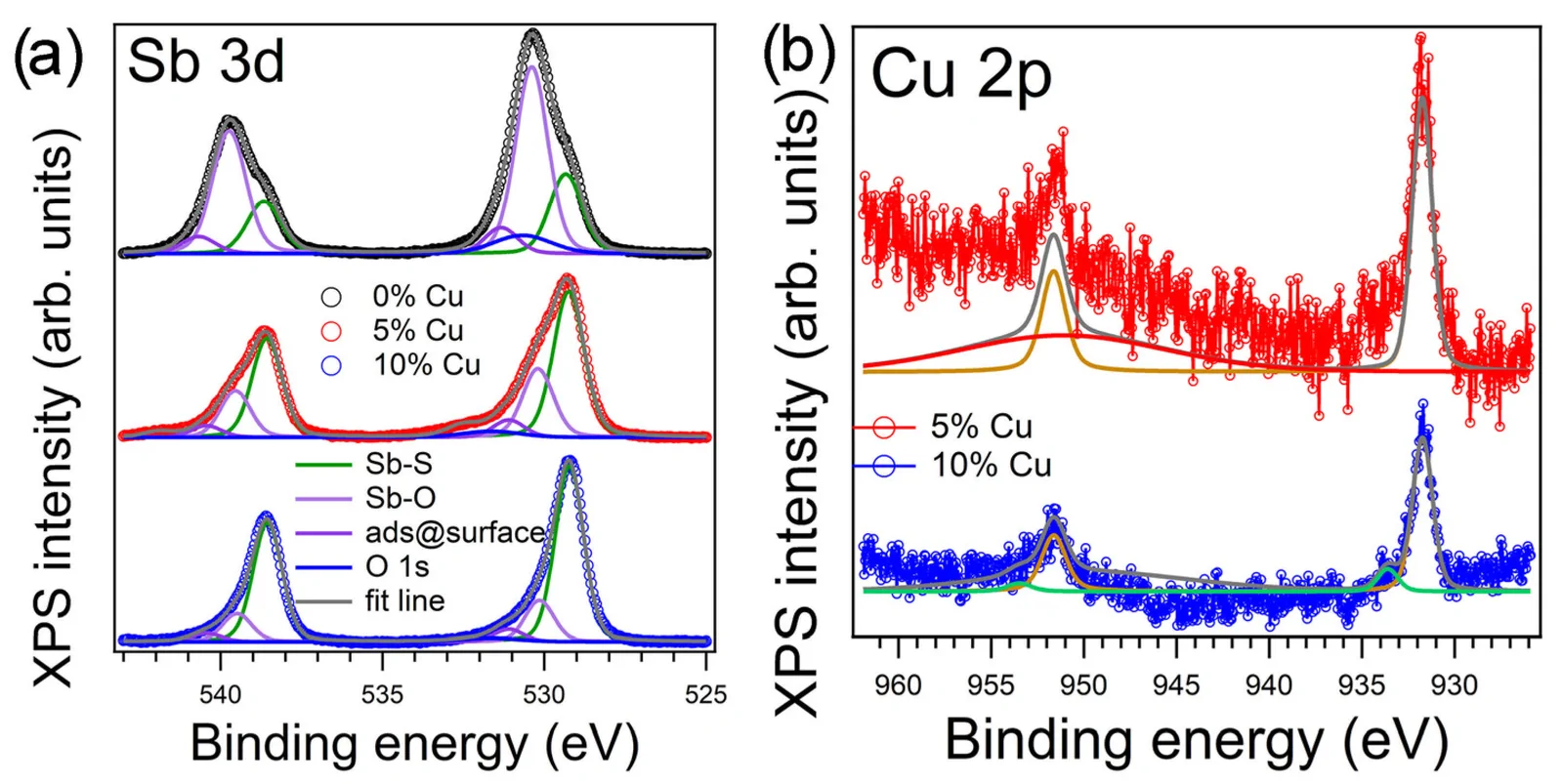
Core XPS spectra of the Sb 3d (**a**) and Cu 2p (fitted with orange line) (**b**) of SS90, C5SS90 and C10SS90 powders.

Survey spectra from XPS analysis of the synthesized powders are presented in Figure S10a, confirming the expected chemical composition. Core spectra of Sb 3d (Figure 20a) show that the undoped sample was highly oxidized and S-deficient, with a S/Sb ratio of 0.47. The Cu-substitution process had a beneficial effect in preventing the surface oxidation of the samples, and the S/Sb ratio got closer to the ideal ratios at 1.35 and 1.47 in C5SS90 and C10SS90, respectively. Interestingly, the Cu content in the surface layer was higher at lower substitution level as can be seen in Figure 20b. As oxides were not detected by XRD or Raman analyses, which possess much larger probing depths, it may be concluded that sulfides from a few of the top surface layers may succumb to oxidation, which should not have a drastic impact on the material as a whole, and this type of defects is often reported for Sb_2_S_3_ [29].

The presence of Cu on the surface of particles is not a true representation of the chemical composition of the bulk samples. When the substitution level was 5%, most of the Cu stayed close to the surface, while at 10% substitution, the distribution of Cu was mostly in the “bulk” of the powders, as can be seen in Figure 20b. This statement is supported by the VB spectra (Figure S10b), where the probing depth of XPS is the highest (considering the valence electrons have the highest kinetic energy). The shoulder, represented by a vertical gray line, shows Cu 4s states. Regarding the oxidation state of Cu 2p, in both the C5SS90 and C10SS90 samples, the dominant oxidation state of Cu is +1, and Cu^2+^ reaches the maximum of 15% in C10SS90.

The surfaces of the doped samples exhibit conductive behavior since the Fermi level is in the conduction band (the intersection of the other grey line represents the VB maxima in Figure S10b). The valence band maximum for Sb_2_S_3_ is at 0.1 eV, and the orange line represents the Fermi level.

### 2.5. Is It Possible to Reach a Pure CuSbS_2_ Composition?

Cu-substituted Sb_2_S_3_ powders contained a CuSbS_2_ secondary phase even when Cu was added in a concentration lower than the stoichiometric amount. This phase is a significant component, so it is more likely for the Sb-Cu-S system to form this phase than for Cu to substitute Sb in Sb_2_S_3_, at least by the synthesis method used here. But, what happens if Cu is added in the same amount as Sb? Would it be possible to obtain pure CuSbS_2_? With similar optical properties to Sb_2_S_3_, this phase is another interesting material for absorbers in photovoltaic devices [12,30]. For this purpose, we tried preliminary syntheses with the temperature, time and stoichiometry modified to get information about their influence on the structure. The synthesis was otherwise conducted in the same way as described in the Section 3.

**Figure 21:**
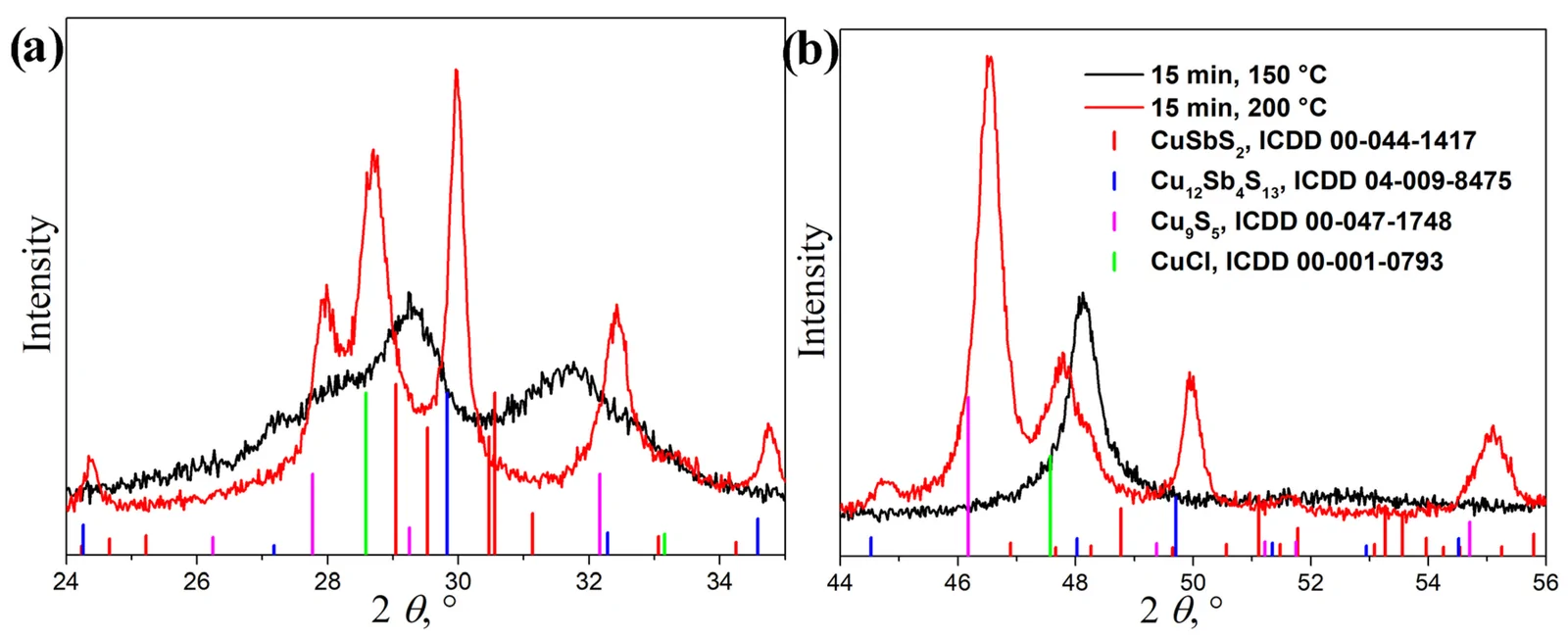
Influence of hot-injection temperature: XRD patterns of CuSbS_2_ samples synthesized for 15 min at 150 °C and 200 °C, presented in two zoomed-in 2*θ* domains: 24–35° (**a**) and 44–56° (**b**).

**Figure 22:**
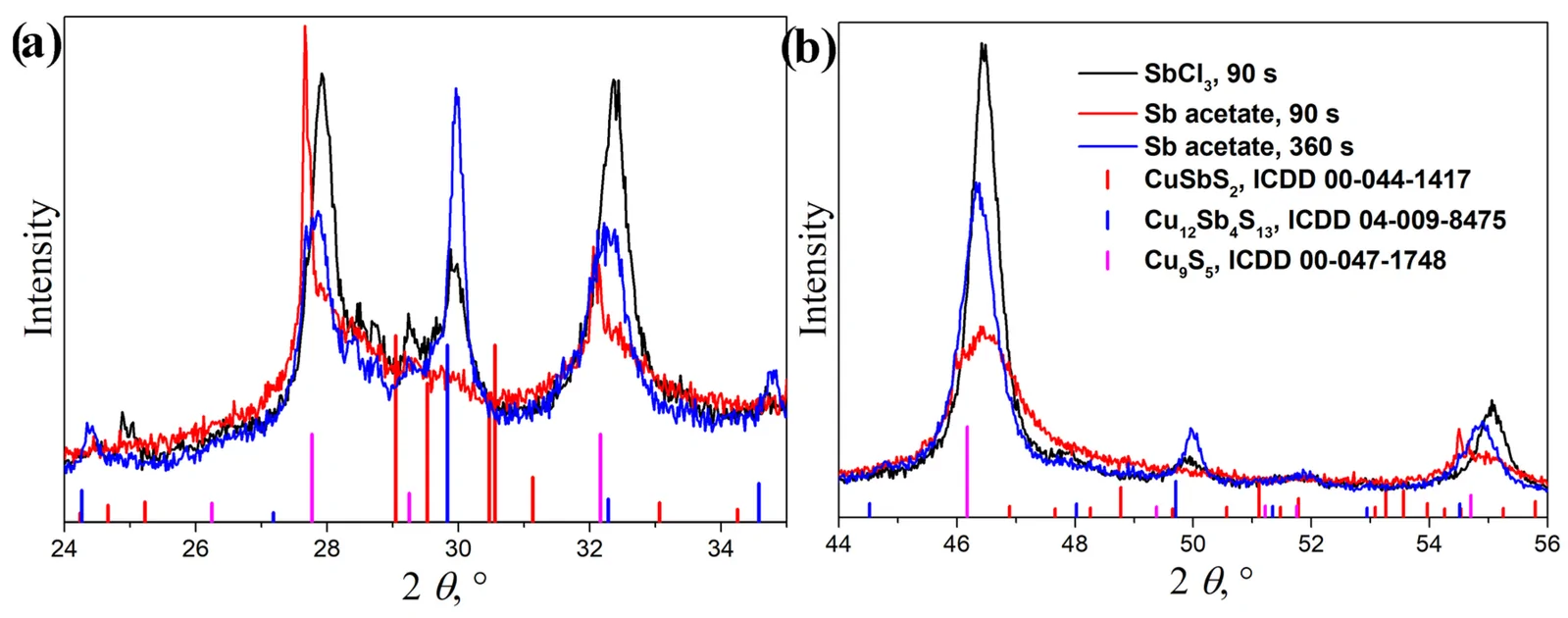
XRD patterns of CuSbS_2_ samples synthesized for 90 s using SbCl_3_, 90 s using Sb acetate and 360 s using Sb acetate, all at 230 °C, presented in two zoomed-in 2*θ* domains: 24–35° (**a**) and 44–56° (**b**).

The kinetics of crystallization is similar to the synthesis of Sb_2_S_3_, meaning nucleation and growth are slow at 150 °C, where 15 min yields a dominantly amorphous product, while the same time at 200 °C allows a clear formation of CuCl, Cu_9_S_5_ and Cu_12_Sb_4_S_13_ as initial crystalline phases (Figure 21). When raising the reaction temperature to 230 °C (as for Sb_2_S_3_), the same level of crystallinity is reached in 1.5 min, but without the formation of CuCl, as can be seen in Figure 22.

Antimony acetate used as a Sb source to prevent the formation of chlorides made the crystallization slower and needed 6 min to develop a similar crystallinity result as SbCl_3_, showing a comparable effect of chloride ions, like that observed during the synthesis of Sb_2_S_3_. The results in Figure 22 imply that the absence of chlorides creates conditions where Cu_9_S_5_ is the phase that initially crystallizes and, over time, it partially recrystallizes into Cu_12_Sb_4_S_13_. This mechanism agrees with the one proposed by the research of Wang et al. identifying copper sulfide acts like nucleation seeds [31].

**Figure 23:**
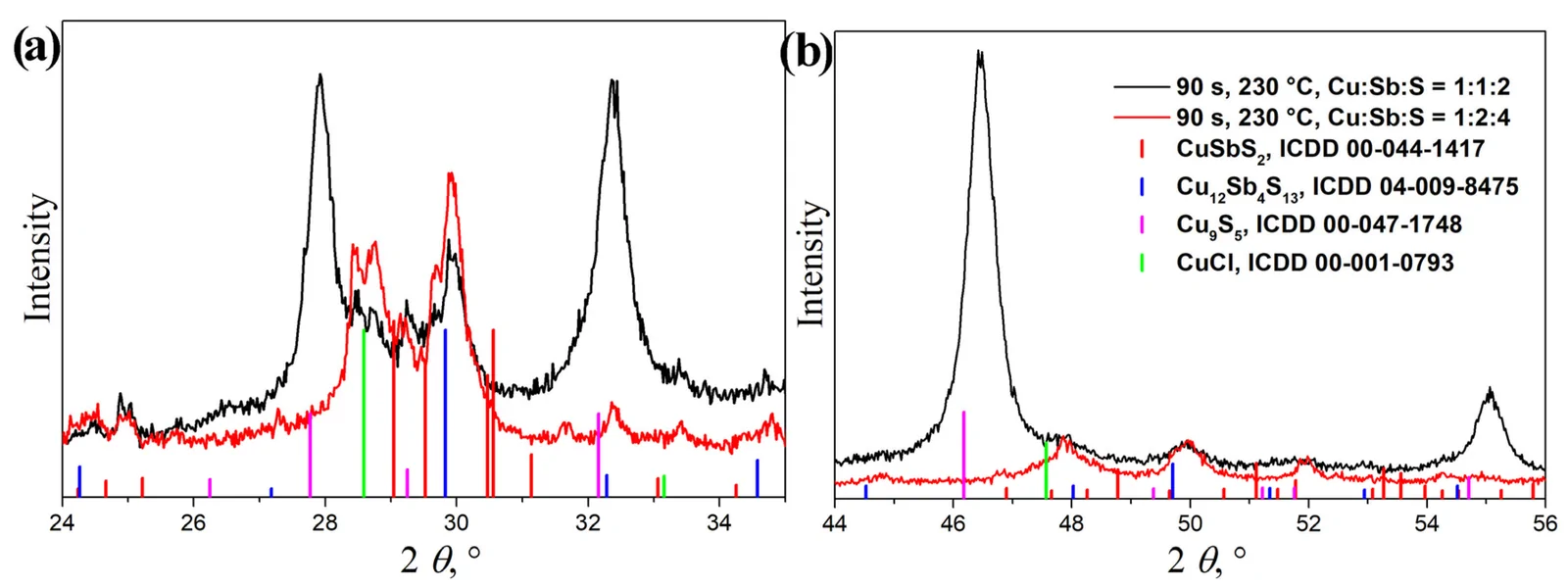
Influence of ion stoichiometry (Cu:Sb:S ratios of 1:1:2 and 1:2:4) on structure, presented in two zoomed-in 2*θ* domains: 24–35° (**a**) and 44–56° (**b**).

It becomes obvious that stoichiometrical composition (Sb:Cu = 1:1) does not produce CuSbS_2_, like it did when the Sb:Cu ratio was 9:1 (10% doping) or 19:1 (5% doping). The Cu_9_S_5_ phase, which initially crystallizes in Cu-rich conditions, prevents the subsequent development of CuSbS_2_. The use of deficient Cu(I) (Sb:Cu = 2:1) prevented the formation of the Cu_9_S_5_ phase, as seen in the diffraction pattern in Figure 23, but only avoided this step to form Cu_12_Sb_4_S_13_ faster, and did not influence the formation of a significant amount of the desired CuSbS_2_ phase at a Cu:Sb:S ratio of 1:2:4.

Structural analysis presented in Figure 21, Figure 22 and Figure 23 shows the formation of several crystalline phases, which were not all identified. The content of the desired CuSbS_2_ phase is very low, while Cu_12_Sb_4_S_13_ and Cu_9_S_5_ are the most prominent. The Cu-Sb-S system produces a lot of possible crystalline structures, and additional optimization is needed to define the conditions needed for the crystallization of the desired product. More preferable conditions for CuSbS_2_ crystallization were present in the non-stoichiometric content of Cu, Sb and S, which existed in 5 or 10% Cu-substituted Sb_2_S_3_ systems with Cu:Sb:S ratios of 1:9:15 and 1:19:30, where almost all added Cu was incorporated into CuSbS_2_. The literature shows that a low Cu content produces a CuSbS_2_ phase [32]. The study by Popovici et al. reported that the optimal Cu(II):Sb:S ratio to produce the highest CuSbS_2_ phase purity (73%) in spray-pyrolysis-synthesized films was 1:3.5:13, meaning that not only a 75% surplus Sb, but also a 550% surplus S was needed [7].

**Figure 24:**
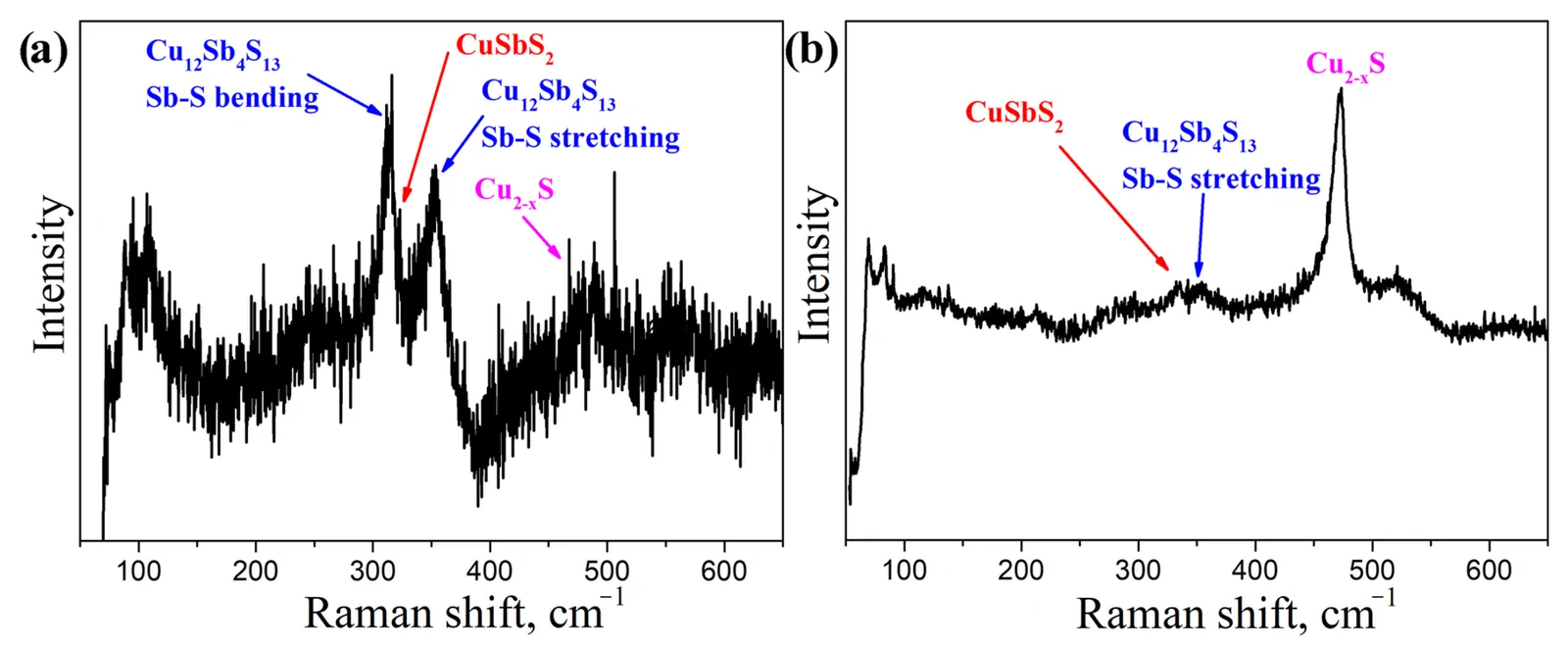
Raman spectra of Sb_2_S_3_ synthesized for (**a**) 15 min at 200 °C (from SbCl_3_) and (**b**) 6 min at 230 °C (from Sb acetate).

Raman analysis of CuSbS_2_ samples confirmed the findings of XRD analysis. After synthesis at 200 °C for 15 min, Sb-S stretching and bending peaks of the Cu_12_Sb_4_S_13_ phase at 350 and 315 cm^−1^ are the most pronounced (Figure 24). It is also possible to notice the Cu_2−x_S peak at 475 cm^−1^ [13,14]. Shorter times of synthesis were too amorphous to exhibit any peaks, and only weak contributions of Cu_12_Sb_4_S_13_ and Cu_2−x_S were noticeable. After 6 min at 230 °C, peaks are also undeveloped, with a dominant Cu_2−x_S phase but, in some regions, it is possible to notice a low presence of the CuSbS_2_ phase (peak at 332 cm^−1^).

**Figure 25:**
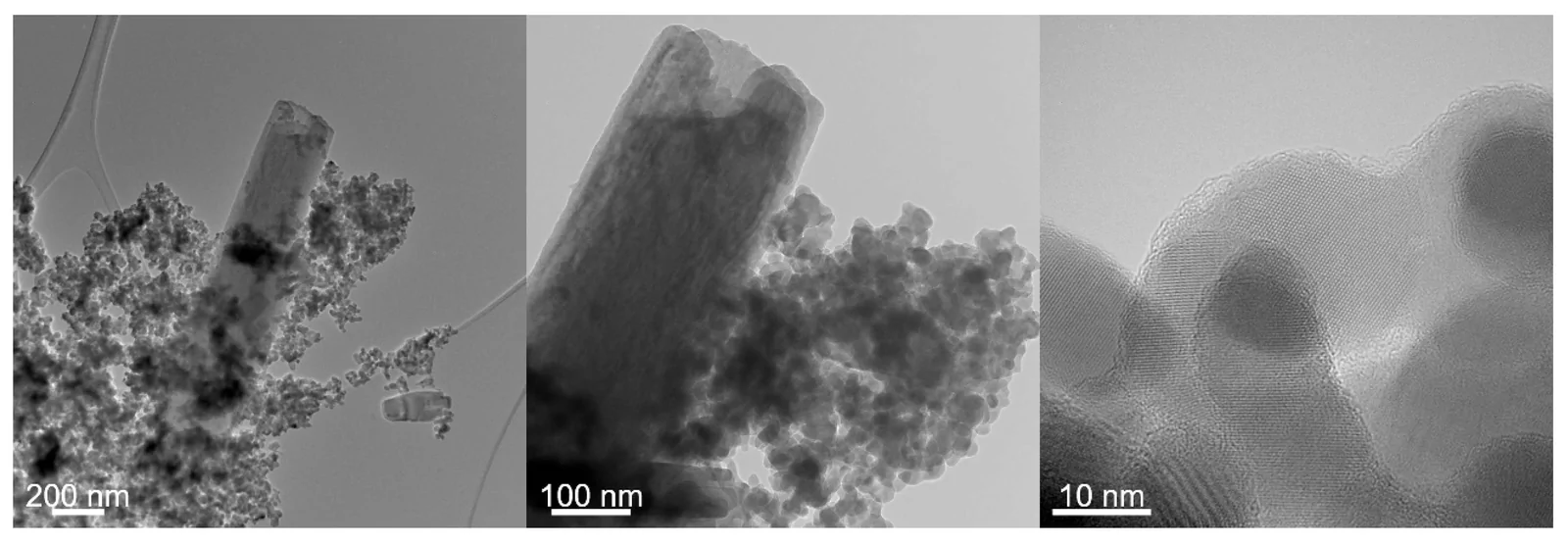
TEM images of CuSbS_2_ powder made from the stoichiometric mixture.

The microstructural analysis of the sample synthesized for 6 min at 230 °C using Sb acetate in the stoichiometric mixture is presented in Figure 25. It shows the powder is composed of rod-like particles longer than 1000 nm and around 300 nm in diameter, and spherical nanoparticles of around 20 nm.

### 2.6. Optical Properties

**Figure 26:**
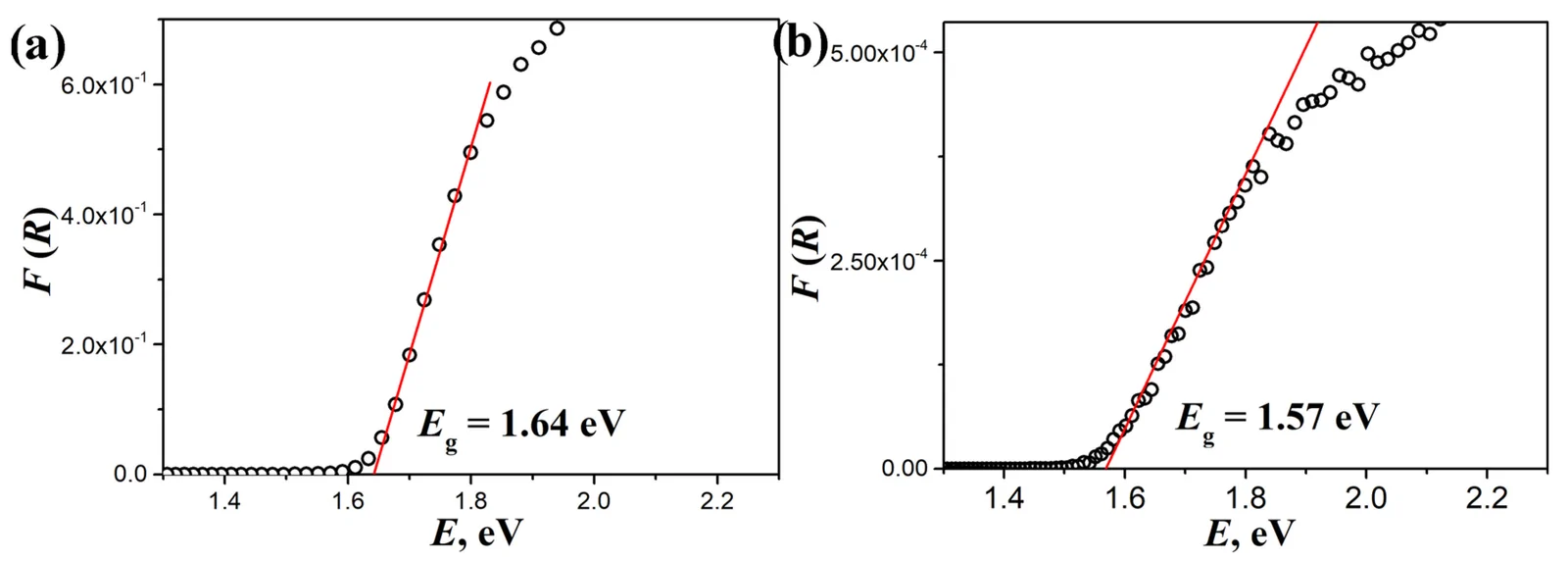
Kubelka–Munk-function fitted direct band gaps of SS90 (**a**) and C10SS90 (**b**).

Optical band gaps of synthesized powders derived from the diffuse reflectance spectra are displayed in Table 1, with the fitted graphs presented for the samples SS90 and C10SS90 in Figure 26. Cu substitution has proven to slightly increase the absorption of the lower-energy spectra, so the band gap energy decreases, which is beneficial for sunlight-driven application. Attempts to synthesize CuSbS_2_ resulted in many phases, resulting in too-complex light-absorption properties to allow band gap determination.

## 3. Materials and Methods

The used starting chemicals were SbCl_3_ (99.0%, Alfa Aesar, Ward Hill, MA, USA), Sb(III) acetate (99.99%, Sigma Aldrich, St. Louis, MO, USA), Cu(I) acetate (97.0%, Sigma Aldrich) and elementary sulfur (99.999%, Alfa Aesar) as sources of antimony, copper and sulfur. Oleylamine (OlA) (≥98.0%, Sigma Aldrich), paraffin oil (Sigma Aldrich, puriss.) and ethylhexanoic acid (EHA) (≥99%, Aldrich Chemistry, St. Louis, MO, USA) were used as solvents, and hexane (95%, J.T. Baker, Phillipsburg, NJ, USA), benzene (99.93%, Lach-Ner, Neratovice, Czech Republic) and isopropanole (99.5%, J.T. Baker) were used to stop the reaction, help with precipitation and redispersion and wash the precipitate. Antimony and copper solutions in EHA preheated to 150 °C were injected into a solution of sulfur in paraffin oil and oleylamine preheated to 240 °C (reaction temperature around 230 °C) and precipitated for 45 or 90 s. After the reaction was stopped by the addition of hexane and isopropyl alcohol, the suspensions were cooled down and precipitated overnight. The precipitate was washed out in benzene, hexane and isopropanol to remove the remaining organics; each washing step was followed by centrifuge treatment for 10 min at 600 rpm. The procedure is explained in more detail in our previous studies [33,34]. The obtained powders were dried at 80 °C for 2 h and ground in an agate mortar. The obtained Sb_2_S_3_ powder samples were named by the substitution level and synthesis time, so 6 samples were present in total, as presented in Table 2: SS45 (Sb_2_S_3_ synthesized for 45 s), SS90 (Sb_2_S_3_ synthesized for 90 s), C5SS45 and C5SS90 (5% Cu(I)-doped Sb_2_S_3_ synthesized for 45 s and 90 s, respectively) and C10SS45 and C10SS90 (10% Cu(I)-doped Sb_2_S_3_ synthesized for 45 s and 90 s, respectively).

X-ray diffraction analysis was conducted using an Automated Multipurpose Powder X-ray Diffractometer XRDynamic 500 by Anton Paar GMBH, Graz, Austria, equipped with a Primux 3000 sealed-tube Cu X-ray source and a Pixos 2000 solid-state hybrid pixel detector. The XRD patterns of the samples were acquired using a Bragg–Brentano geometry in the continuous mode with a scan speed of 0.02°s^−1^ in a 2*θ* range from 10° to 140°.

Raman analysis was done employing a LabRAM HR Evolution Raman spectrometer (Horiba Jobin-Yvon, Montpellier, France) with a confocal microscope and a He–Ne laser at an excitation wavelength of 633 nm.

The microstructure and elemental distribution of the nanoparticles were analyzed using a JEOL-F200CF STEM apparatus (JEOL Ltd., Tokyo, Japan) equipped with an EDS system. A diluted particle solution was drop-casted onto nickel grids.

The high-resolution spectra for all of the core levels were measured by X-ray photoelectron spectroscopy (XPS) using a Kratos Ultra DLD spectrometer (Kratos Analytical, Manchester, UK) with a monochromatized Al Kα source (1486.6 eV). The source was operated at 144 W (12 mA and 12 kV), with 20 eV and 50 meV energy step resolutions. The charging effects were compensated using a flood gun and a correction of the binding energies to C=C contamination at 284.8 eV.

Optical properties of the synthesized powders were tested using a Shimadzu UV-2600 spectrophotometer (Shimadzu Corporation, Kyoto, Japan) equipped with an integrated sphere, measuring the reflectance of the layer of powder sample on a barium sulphate bed. Band gap energies were calculated using the Kubelka–Munk approximation (https://mmrc.caltech.edu/FTIR/Literature/Diff%20Refectance/Kubelka-Munk.pdf (accessed on 12 May 2026)).

## 4. Conclusions

Hot-injection synthesis was employed to synthesize copper-doped Sb_2_S_3_ nanoparticles of different morphologies than typical elongated rods of Sb_2_S_3_. Obtained powders were characterized to define the influence of the presence of Cu on their structure, microstructure and optical properties. Structural analysis implied that the metallic nature of Cu was too different from metalloid Sb in predominantly covalent Sb-S bonding to successfully substitute it in the Sb_2_S_3_ structure, despite suitable ionic radii. Elemental analysis of Cu-substituted samples revealed the spatial distribution of S, Sb and Cu uniformly distributed in all particles, with Cu-rich regions of the CuSbS_2_ phase existing without a change in S and Sb distributions. This contradicts the XRD results that show the substitution of Sb by Cu does not take place. One explanation may be that Cu is incorporated into the Sb_2_S_3_ phase on Sb positions, but sizes of Sb and Cu are similar enough to not disturb the structure at all. Another option is that Cu present in the amorphous phase uniformly covers the surface of Sb_2_S_3_ particles, showing a homogeneous distribution of Cu in those regions and a higher content in the particles of in CuSbS_2_ phase. The dominant microstructure of elongated rod-like particles of Sb_2_S_3_ and minor spherical amorphous nanoparticles remained the same in Cu-substituted samples, with the addition of nanoplatelets of CuSbS_2_. Cu substitution decreased the direct band gap, improving the absorption of the visible spectra. Different stoichiometries of the reactants were tested in the same synthesis method with the idea to obtain a pure CuSbS_2_ phase, which is another potential absorber material in photovoltaics, but these conditions were less favorable for the crystallization of the desired material. The precipitation of Cu is significantly faster than that of Sb, so excesses of Sb and S are needed to produce the CuSbS_2_ phase, creating a lot of unreacted dissolved ions in the liquid phase. The poor solubility of copper chlorides in organic solvents introduced another secondary phase, which may be prevented by using Sb acetate as the source of Sb, though the absence of chlorides also has a retarding impact on the kinetics of precipitation. Our future work should optimize synthesis conditions and reactant ratios to explore two possible directions of research: (1) the production of crystalline Sb_2_S_3_ particles with a reduced aspect ratio, and (2) the production of pure CuSbS_2_ powder, both to be tested as a light absorber in photovoltaic devices.

## Figures and Tables

**Table 1:** Direct and indirect band gaps of synthesized materials.

Sample	*E*_g_, Direct, eV	*E*_g_, Indirect, eV	Direct/Indirect
SS45	2.04	1.79	Indirect
SS90	1.64	1.52	Direct
C5SS90	1.67	1.50	Direct
C10SS90	1.57	1.42	Direct
“CuSbS_2_”	Unreliable, ~2.0		Direct

**Table 2:** Preview of the samples based on synthesis time and copper substitution level.

Time, s	Copper Substitution Level, mol %		
	0	5	10
45	SS45	C5SS45	C10SS45
90	SS90	C5SS90	C10SS90

## Data Availability

The raw data supporting the conclusions of this article will be made available by the authors on request.

## Supplementary Materials

The following supporting information can be downloaded at: https://www.mdpi.com/article/10.3390/ijms27167406/s1.
