# Supplementary material for: Cu-Substituted Sb2S3 Nanopowders—Structural, Microstructural and Optical Analyses
Source: Int J Mol Sci. 2026 Aug 19;27(16):7406. doi: 10.3390/ijms27167406 (PMC13513189; doi:10.3390/ijms27167406)
Supplement: Supplementary file 1 [file ijms-27-07406-s001.zip › ijms-4463143-supplementary.pdf]

Supporting Information

Cu substituted Sb_2S_3 nanopowders – structural, microstructural and optical analysis

Nikola Ilić[1], Cristian Radu[2], Amelia Elena Bocirnea[2], Aurelian Catalin Galca[2], Ivana Validžić[1]

Additional XRPD data, Raman analysis, HRTEM micrographs and EDS analysis, as well as STEM-DF images.

Content:

1. Additional XRPD micrographs

2. Additional Raman analysis

3. Additional HRTEM micrographs

4. Additional EDS analysis

5. STEM-DF images

6. XPS survey spectra

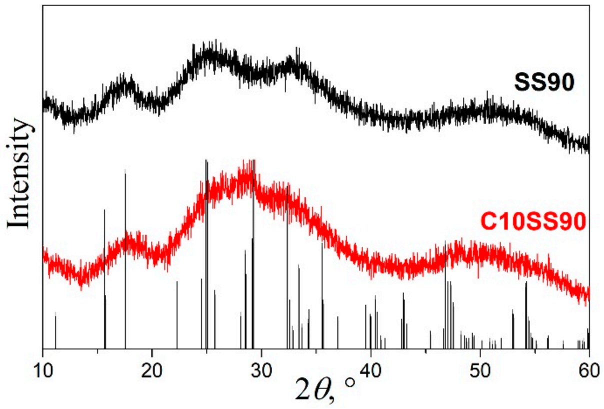

Figure S1. XRD patterns of Sb_2S_3 and 10 % Cu-substituted Sb_2S_3 powders synthesized for 90 s using Sb-acetate as Sb source.

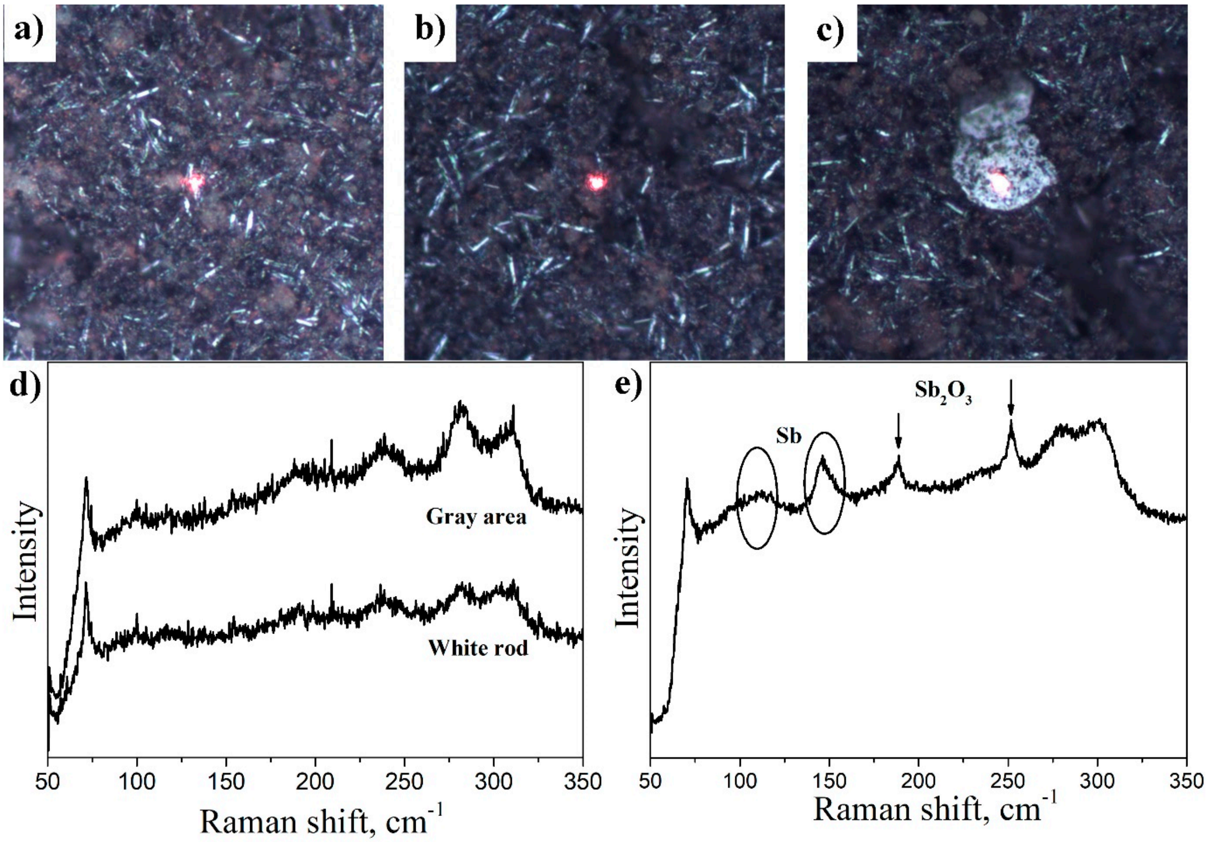

Figure S2. Appearance of SS90 powder under confocl microscope: a) white rods, b) gray areas in-between, with c) corresponding Raman spectra of both regions.

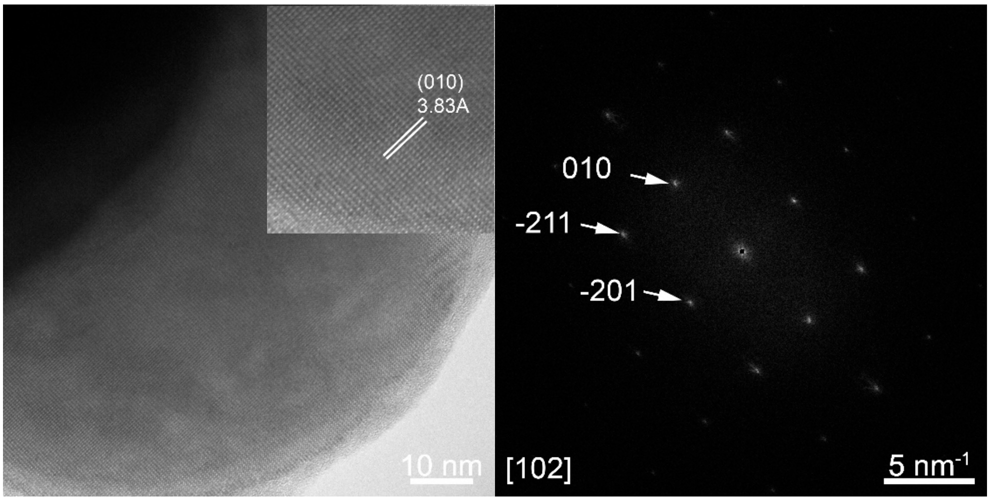

Figure S3. HRTEM and Fourier transform of the microrod particle in SS45 sample.

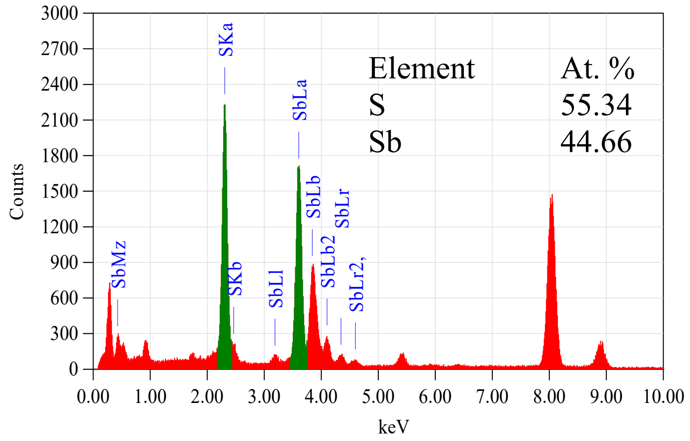

Figure S4. EDX spectrum and chemical composition of the area in Figure 9b.

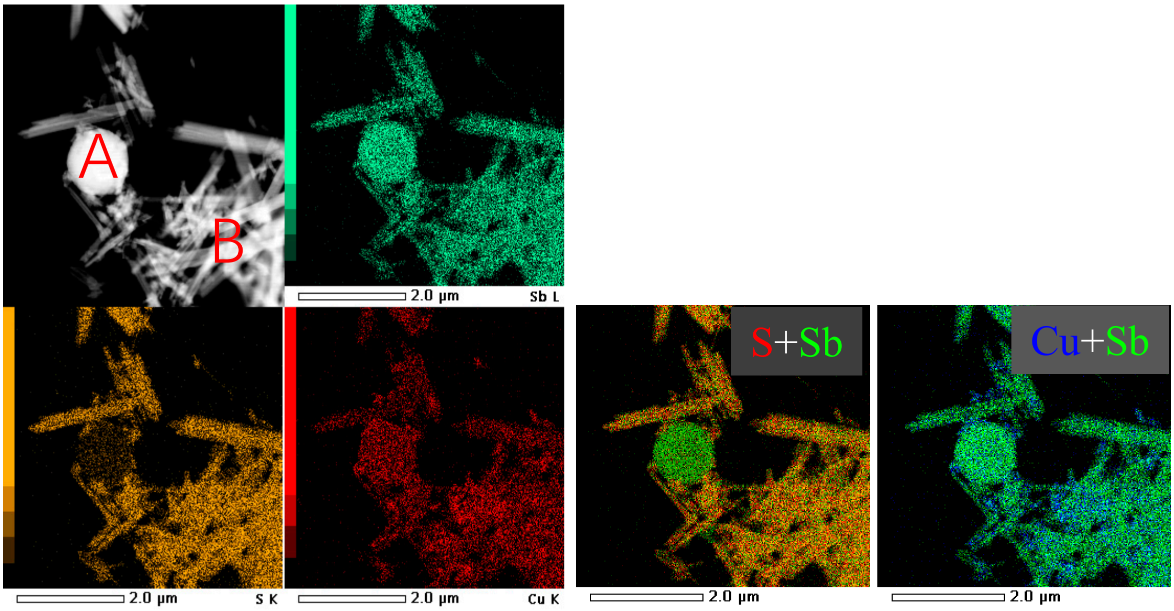

Figure S5. Mapping of the C5SS90 (a) with S/Sb distribution overlap (b) and Cu/Sb distribution overlap (c).

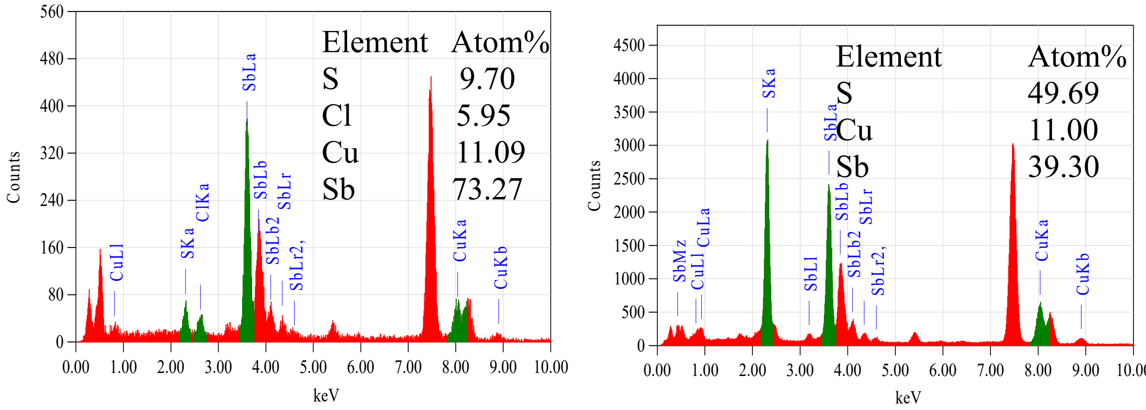

Figure S6. EDX spectrum and chemical composition of the areas A (a) and B (b) in Figure S5.

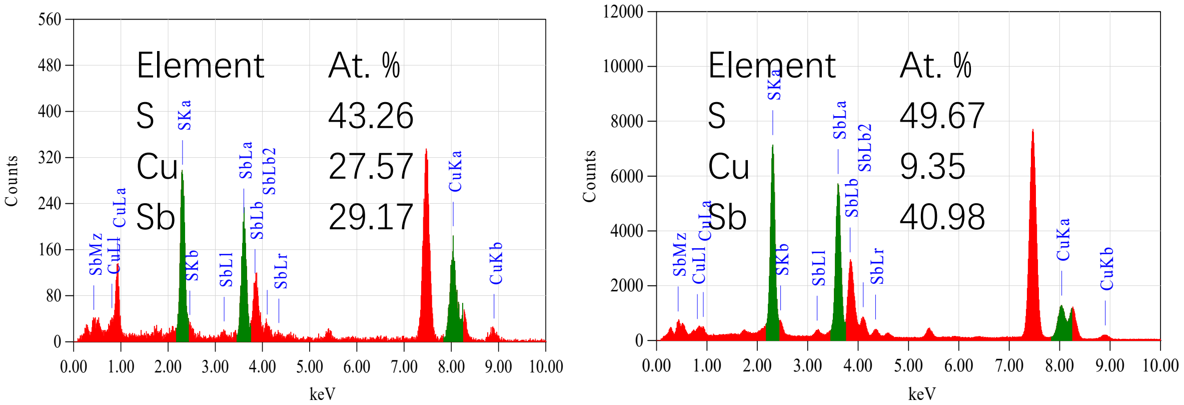

Figure S7. EDX analysis of regions: a) A and b) B from Figure 13.

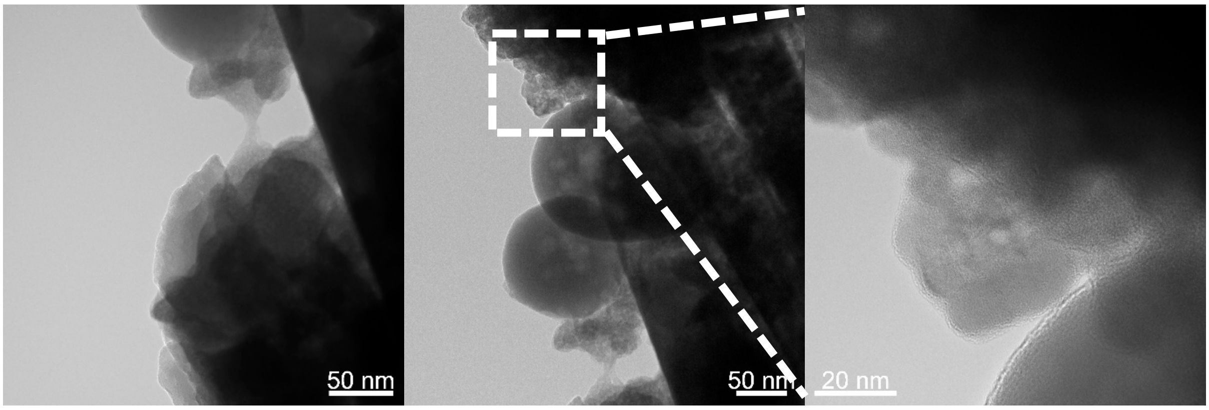

Figure S8. TEM images of amorphous phase accompanying the Sb_2S_3 rods described as type 3 morphology of amorphous spherical nanoparticles.

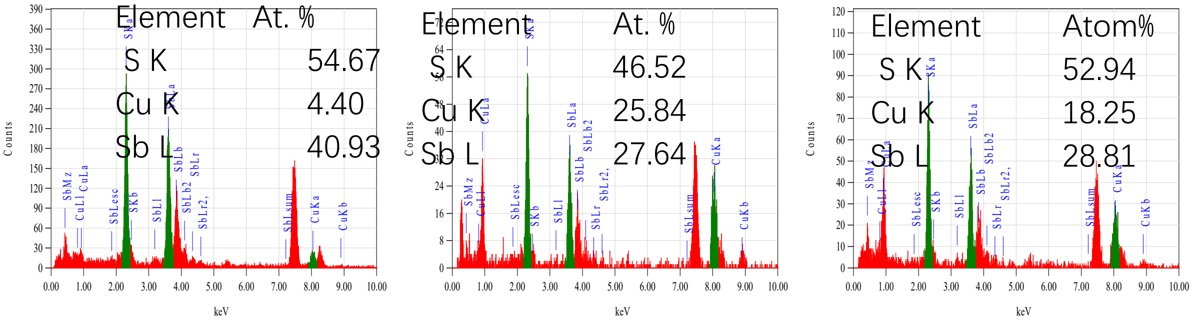

Figure S9. EDX analysis of regions A, B and C from Figure 18.

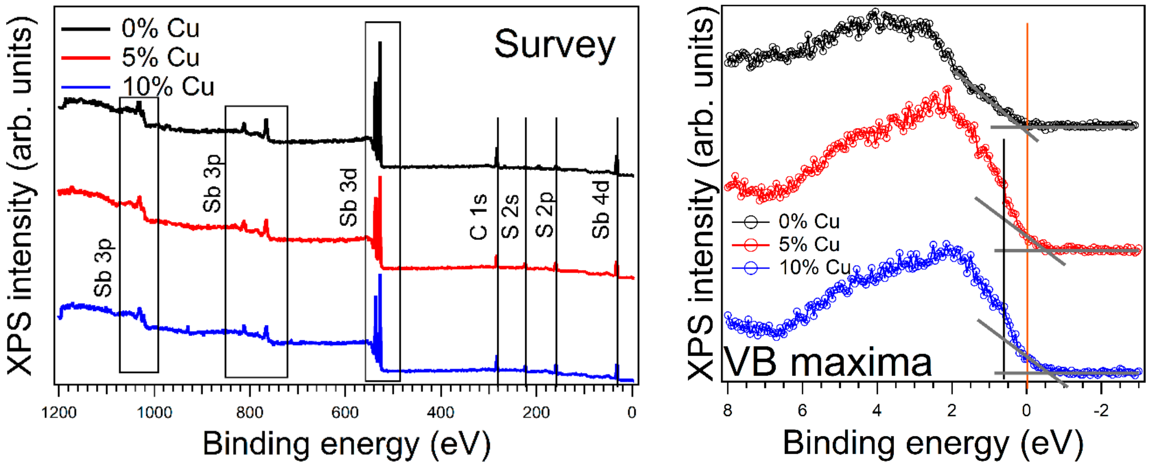

Figure S10. XPS survey spectra (a) and VB spectra (b) of SS90, C5SS90 and C10SS90 powders.
